# Genomic insights into local adaptation in the Asiatic toad *Bufo gargarizans*, and its genomic offset to climate warming

**DOI:** 10.1111/eva.13555

**Published:** 2023-05-02

**Authors:** Lu‐Wen Zhang, Jun‐Qiong Chen, Ru‐Meng Zhao, Jun Zhong, Long‐Hui Lin, Hong Li, Xiang Ji, Yan‐Fu Qu

**Affiliations:** ^1^ College of Life Sciences Nanjing Normal University Nanjing China; ^2^ Zhejiang Provincial Key Laboratory for Water Environment and Marine Biological Resources Protection, College of Life and Environmental Sciences Wenzhou University Wenzhou China; ^3^ College of Life and Environmental Sciences Hangzhou Normal University Hangzhou China

**Keywords:** Bufonidae, genomic offset, global warming, isolation, population structure, SNP

## Abstract

Genomic signatures of local adaptation have been identified in many species but remain sparsely studied in amphibians. Here, we explored genome‐wide divergence within the Asiatic toad, *Bufo gargarizans*, to study local adaptation and genomic offset (i.e., the mismatch between current and future genotype‐environment relationships) under climate warming scenarios. We obtained high‐quality SNP data for 94 Asiatic toads from 21 populations in China to study spatial patterns of genomic variation, local adaptation, and genomic offset to warming in this wide‐ranging species. Population structure and genetic diversity analysis based on high‐quality SNPs revealed three clusters of *B. gargarizans* in the western, central‐eastern, and northeastern portions of the species' range in China. Populations generally dispersed along two migration routes, one from the west to the central‐east and one from the central‐east to the northeast. Both genetic diversity and pairwise *F*_ST_ were climatically correlated, and pairwise *F*_ST_ was also correlated with geographic distance. Spatial genomic patterns in *B. gargarizans* were determined by the local environment and geographic distance. Global warming will increase the extirpation risk of *B. gargarizans*.

## 1 INTRODUCTION

Clarifying the genomic signatures, genes, and traits underlying local adaptation of organisms to their environments is a major goal in evolutionary biology. Local adaptation can generate genetic differentiation and divergence among populations, which may lead to reduced gene flow and eventually speciation (Merilä et al., 2004; Savolainen et al., 2013). Local adaptation across a landscape can disrupt patterns of isolation‐by‐distance (IBD) and result in patterns of isolation‐by‐environment (IBE; Sexton et al., 2014; Wang & Bradburd, 2014). Isolation‐by‐resistance (IBR), a modification of IBD, predicts that patterns of genetic divergence are explained by geographic distances accounting for variation in the degree of resistance to dispersal that organisms experience through heterogeneous landscapes (MacDonald et al., 2020; McRae, 2006). Studies of patterns of IBE, IBR, and IBD have facilitated our understanding of local adaptation in various organisms, such as the swallowtail butterfly *Papilio machaon dodi* (MacDonald et al., 2020) and *Anolis* lizards (Wang et al., 2013), but the trend of genetic diversity under the background of global change still remains a sparsely studied area.

Most studies of local adaptation at the molecular level have focused on identifying candidate genes and characterizing their functions (Tiffin & Ross‐Ibarra, 2014). Nevertheless, a few studies have been done to compare current and future genotype‐environment mismatches and thereby explore the extent of genomic offset to climate change (Cummins et al., 2019). Genomic offset is by definition the genetic distance between the present and the predicted genomic composition, with a smaller genomic offset translated into a lower risk of population decline (Rellstab et al., 2021). Many single‐nucleotide polymorphism (SNP) loci and candidate genes closely related to environment variables have been identified in a diverse array of taxa including several species of anuran amphibians such as *Pseudophryne guentheri* (Cummins et al., 2019), *Rana temporaria* (Bonin et al., 2005), and *Nanorana parkeri* (Wang et al., 2018). These SNPs and genes have potential impact on the survival of organisms in nature. In recent years, increasingly more studies have used SNP datasets to visualize current and future genomic landscapes (Fitzpatrick & Keller, 2015; Ingvarsson & Bernhardsson, 2020). These studies not only provide key insights into how organisms adapt to their environments at the genomic level but also contribute to better coping with the impact of future environmental change from the perspective of biological conservation.

Amphibians have unique habitat requirements and life‐history characteristics, making them more vulnerable to climate change compared with other terrestrial vertebrates (Ficetola et al., 2015). More than 8000 species of amphibians have been described, and many populations are on the decline and even have been extirpated from their natural habitats (Baillie et al., 2004; Holt et al., 2013; Nyström et al., 2007). The global decline of amphibian populations is driven by several factors, including habitat fragmentation (Marsh & Trenham, 2001), human activities (Nowakowski et al., 2018), infectious disease (Densmore & Green, 2007), biological invasions (Mazzoni et al., 2003), and climate change (Pounds et al., 2006). The effects of climate change on individual species ultimately depend on their physiological tolerance and dispersal ability (Cummins et al., 2019; Lawler et al., 2010; Qu & Wiens, 2020).

China exhibits complex geomorphological and climatic characteristics. The altitudinal gradient (low in the east and high in the west) has a substantial effect on the distribution and genetic patterns of organisms (Huang et al., 2013; Qu et al., 2014; Wu et al., 2016). Numerous mountain ranges, deep valleys, and rivers make the topography of China particularly complex and provide opportunities for populations to become geographically isolated. For example, geological and climatic events have contributed to the current geographic distributions of frogs of the genera *Babina*, *Nanorana*, *Pelophylax*, *Quasipaa*, *Rana*, and *Rhacophorus* (Che et al., 2010; Li et al., 2012, 2015; Liu et al., 2015; Yang et al., 2017; Zhang et al., 2008). Geographic barrier has also been shown to be a key factor determining spatial patterns of genetic variation in several species of anurans such as *Feirana quadranus* (Wang et al., 2012), *Quasipaa boulengeri* (Yan et al., 2013), *Odorrana schmackeri* (Li et al., 2015), *Nanorana pleskei* (Zhou et al., 2017), and *Leptobrachium ailaonicum* (Zhang et al., 2009). Studies of the genomic offset to climate warming at the population level can provide key insights into how patterns of biodiversity vary in a region in response to climate change; however, such studies have been rarely conducted.

The Asiatic toad (*Bufo gargarizans*) is common throughout its range in China, part of Russia, Japan, and the Korean Peninsula (Fu et al., 2005; Zhan & Fu, 2011). Its wide distribution spanning nearly 20 degrees of latitude with high environmental heterogeneity makes it an excellent model for studying the effects of environmental and geographical factors on the genetic structure of populations (Fu et al., 2005; Macey et al., 1998; Pan et al., 2018; Tong et al., 2017; Wu & Hu, 2010; Zhan & Fu, 2011). Previous phylogenetic studies of *B. gargarizans* using mitochondrial and nuclear markers have suggested that vicariance, repeated range expansions and heterogeneous landscapes have played key roles in shaping the genetic structure of populations (Fu et al., 2005; Pan et al., 2018; Tong et al., 2017; Zhan & Fu, 2011). However, quantitative analyses of the contribution of geographical and environmental factors to the formation of genetic patterns in *B. gargarizans* have not yet been conducted. Such analyses are of importance in predicting the impact of future environmental change on *B. gargarizans* populations and developing effective protection strategies for this species that has undergone rapid population decline and even local extinction in China.

Over the past decade, genome‐wide SNP datasets have been increasingly used as molecular markers to study the genetic pattern and diversity of organisms for the following reasons. First, SNP datasets are cost‐effective, allow sampling schemes to cover a broader geographical distribution range, and more reliably evaluate genetic patterns and variation at different spatial scales (Anderson et al., 2010; Maigret et al., 2020). Second, using SNP datasets can reduce the number of individuals required for quantifying differences among sampling locations (Nazareno et al., 2017). Third, SNP datasets are useful in detecting weak spatial genetic patterns, thus allowing better implementation of genetic conservation practices (Maigret et al., 2020). Here, we conducted a genome‐wide analysis of genetic divergence within *B. gargarizans* using landscape genetic approaches. Specifically, we aimed to (1) identify the climatic and geographic variables contributing to genomic divergence and local adaptation in *B. gargarizans*, (2) determine how climate change will affect future patterns of genetic diversity among populations, and (3) evaluate the genomic offset of *B. gargarizans* to climate change across its range.

## 2 MATERIALS AND METHODS

### 2.1 Sampling and DNA extraction

**FIGURE 1:**
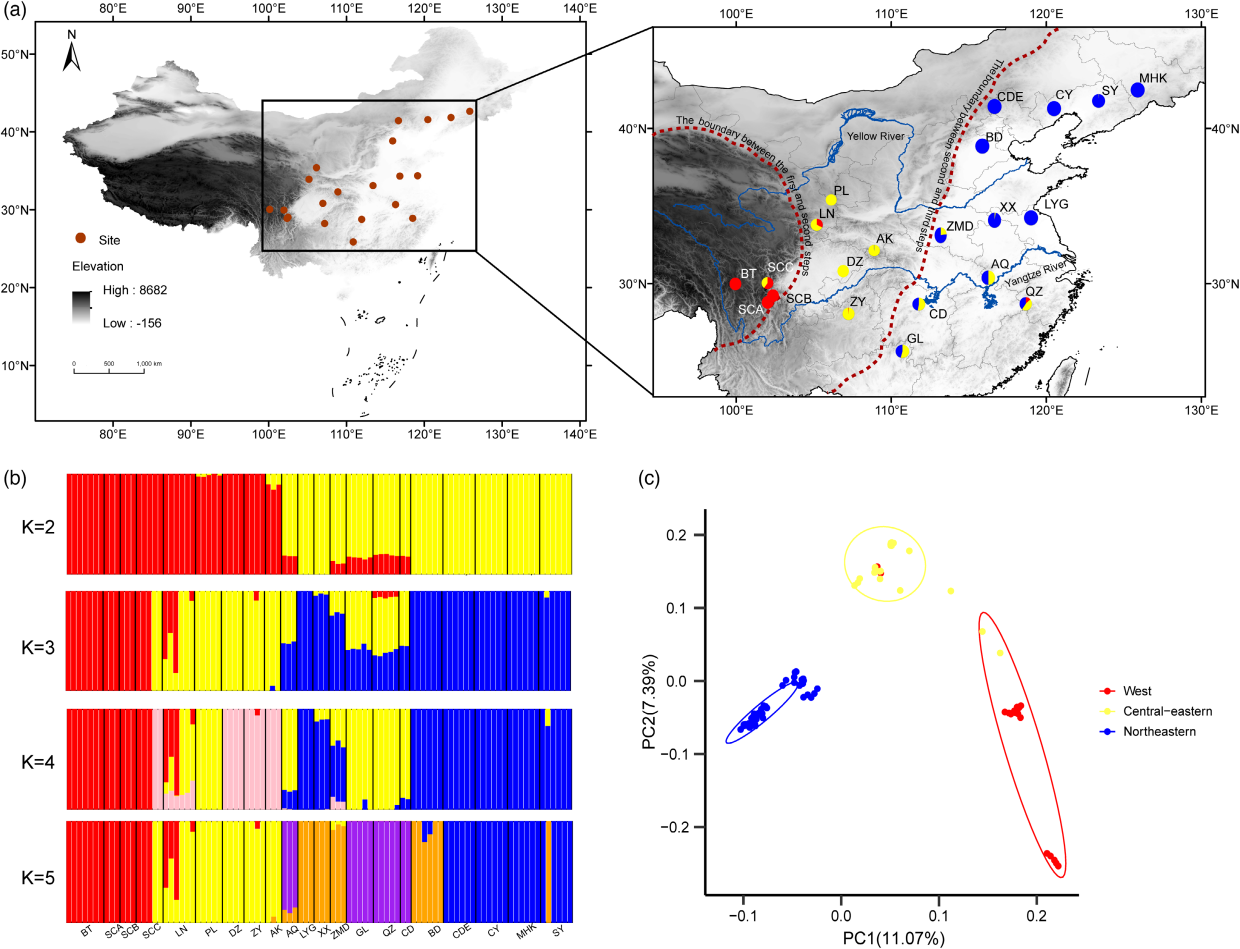
Map of China, showing the 21 sampling localities (brownish red dots) and the important geographic barriers including the Yellow River, the Yangtze River, the boundary between the third and second terrain steps, and the boundary between the second and first terrain steps (a). Results of the ADMIXTURE analysis of *B. gargarizans* samples (K = 2–5, with an optimal K of 3) based on high‐quality loci (b). Results of PCA (c), each dot represents an individual, and the ovals represent 85% confidence intervals for different evolutionary clusters. AK: Ankang, Shanxi; AQ: Anqing, Anhui; BD: Baoding, Hebei; BT: Litang, Sichuan; CD: Changde, Hunan; CDE: Chengde, Hebei; CY: Chaoyang, Liaoning; DZ: Dazhou, Sichuan; GL: Guilin, Guangxi; MHK: Meihekou, Jilin; LN: Longnan, Gansu; LYG: Lianyungang, Jiangsu; QZ: Quzhou, Zhejiang; PL: Pingliang, Gansu; SCA: Shimian2, Sichuan; SCB: Shimian2, Sichuan; SCC: Kangding, Sichuan; SY: Shenyang, Liaoning; XX: Xiaoxian, Anhui; ZMD: Zhumadian, Henan; ZY: Zunyi, Guizhou.

We obtained muscle tissue samples from 94 adult Asian toads collected in 2018 and 2019 from 21 populations covering the entire range of *B. gargarizans* in China, with altitudes varying from 2 to 4006 m above sea level and between‐population distances from 21 to 2846 km (Figure 1a). Although four populations (SCA, SCB, SCC, and BT; see Table S1 for detailed population information) in western China are in close geographic proximity, the altitudinal difference between populations is not smaller than 500 m (Figure 1a). The sampling area is characterized by a high degree of environmental heterogeneity, with annual mean temperature and annual precipitation both decreasing from south to north and annual precipitation also decreasing from east to west. Our sampling localities included but were not limited to those reported in previous studies of the genetic diversity, population genetic structure, and phylogeography of *B. gargarizans* based on mitochondrial DNA (Fu et al., 2005; Hu et al., 2007), microsatellite (Pan et al., 2018; Wu & Hu, 2010), or nuclear and mitochondrial DNA data (Wen et al., 2015; Zhan & Fu, 2011).

Tissue samples were brought to our laboratory in Nanjing, where we used dBIOZOL Genomic DNA Extraction Reagent (Bioer Technology) to extract total genomic DNA from each sample. The quality of genomic DNA was assessed by 1% agarose gel electrophoresis. DNA templates were stored at −80°C for later use.

### 2.2 Sequencing, quality control, and SNP‐calling

We used the double‐digest restriction site‐associated DNA sequencing method to develop genome‐wide SNP markers (Peterson et al., 2012). Libraries were constructed for each sample per the steps provided by Sangon Biotech Co., Ltd. First, genomic DNA was double‐digested with restriction enzymes. The double‐digest reactions were carried out in a volume of 30 μL containing 250 ng of genomic DNA, 3.2 U *Mse*I (New England BioLabs, R0106), 0.8 U *Pst*I‐HF (New England BioLabs, R3140), and 10× cut smart buffer. The reaction mixture was incubated at 37°C for 6 h and 65°C for 90 min. Second, the ligation reaction between fragments and adaptors was conducted in a reaction volume of 40 μL at 22°C for 2 h, containing 0.1 μM *Mse*I adaptors, 15 μM common adaptors, and 200 U/μL T4 DNA Ligase (New England Biolabs, M0202). Third, each sample was amplified by PCR in a 25‐μL reaction volume containing 50–100 ng of adaptor‐ligated DNA fragments as template, 5 μL of 5× Master Mix (New England BioLabs, M0285), and two overhang primers (5 μM). PCR amplification was performed using the following program: 95°C for 30 s; 16 cycles at 95°C for 30 s, 60°C for 20 s, and 68°C for 15 s; and a final extension at 65°C for 5 min. The PCR products were run on a 1.5% agarose gel, and 300‐ to 500‐bp fragments were recovered from the gel. All samples were combined in a single pool for library construction. The quantity of DNA in the libraries was determined using a Qubit fluorometer (Invitrogen), and libraries were considered qualified when the DNA concentration was greater than 5 ng/μL. The qualified libraries were sequenced on the Illumina Novaseq PE150 platform using 150‐bp pair‐end reads. A total of 5 Gb of raw sequencing reads were obtained for each sample in this study. These raw sequencing reads have been deposited in the National Genomics Data Center (NGDC) GSA database (accession number CRA005915).

The Illumina sequencing data were evaluated using FastQC 0.11.8 (Kircher, 2012), and trimmed by Trimmonatic 0.39 (Bolger et al., 2014) to obtain accurate and valid data. We filtered the original data by removing sequences with N bases, adapter sequences, low‐quality bases from the forward and reverse sequences (Q value < 20), and reads with lengths less than or equal to 35 nt. A sliding window trimming was performed when the average quality was below a threshold of window size (5) and required quality (20).

Using Burrows‐Wheeler Aligner 0.7.17 (BWA‐MEM; Li & Durbin, 2009), we aligned high‐quality reads to a *B. gargarizans* reference genome consisting of 11 chromosomes, of which the sizes ranged from 100.36 to 759.86 Mb, with a total genome length of 5.4 Gb (NCBI with a BioProject Accession number of PRJNA628553). We used SAMtools 1.11 to convert SAM files to BAM files (Danecek et al., 2021) and Picard 2.8.3 to remove repeated reads using “MarkDuplicates” for all individuals (Koike & Shiga, 2007). The total variant call format (VCF) file containing SNP loci across all samples was obtained using GATK's Haplotype Caller 4.0 and used for variant discovery (McKenna et al., 2010). VCFtools 0.1.16 was used to filter SNP data with the parameters of ‐minQ 30, ‐maf 0.01, and ‐max‐missing 0.7 (Danecek et al., 2011).

### 2.3 Population genetic structure and diversity

We used two independent multivariate and model‐based methods to evaluate the genetic structure of populations. First, we used ADMIXTURE 1.4, a model‐based clustering program, to characterize population structure (Alexander et al., 2009). The ADMIXTURE analysis was conducted with *K* values ranging from 2 to 10, and the optimal number of groups was the *K* value with the lowest cross‐validation error. We then used the package Plink 1.9 to perform principal components analysis (PCA) based on the high‐quality SNP data and thereby analyzed population genetic structure (Chang et al., 2015). The number of clusters among populations was assessed using the *K*‐means clustering method for ADMIXTURE analysis and the number of PC axes that minimized the Bayesian information criterion score for PCA. We used the package ggplot2 in R (R Development Core Team, 2020) to visualize the PCA results.

The GTRGAMMA nucleotide substitution model was used in RAxML 8.2.9 (Stamatakis, 2014) to construct a maximum likelihood (ML) phylogenetic tree based on high quality SNPs. The pairwise mean population differentiation (*F*_ST_) was calculated using the high‐quality SNPs in VCFtools. Genetic diversity, including nucleotide diversity (π), average expected heterozygosity (*H*e), average observed heterozygosity (*H*o), and Wright's inbreeding coefficient (*F*_IS_), was measured using the program Populations in Arlequin 3.5.2.2 (Excoffier & Lischer, 2010). Genetic diversity at the population level was assessed using the 21 sampled populations (Table S1).

### 2.4 Habitat suitability

We used Maxent to calculate resistance surface parameters to map the habitat suitability of *B. gargarizans* in our study landscape (Phillips et al., 2006). A total of 356 occurrence data were obtained from the 21 populations in this study, and 335 georeferenced records downloaded from the Global Biodiversity Information Facility (Table S2; GBIF.org, 2020). We extracted the 23 environmental variables for GIS data layers obtained from related websites, including 19 bioclimatic variables, solar radiation (SR), mean annual actual evapotranspiration (AET), and altitude from the Worldclim data website (Fick & Hijmans, 2017), normalized difference vegetation index (NDVI, data source: https://earthdata.nasa.gov), and mean annual solar radiation (Wm‐2, SR, data source from 1961 to 1990: https://earthdata.nasa.gov). These environmental variables were selected based both on their perceived biological relevance for the distribution of this toad species and the low degree of collinearity between variables (Giovannini et al., 2014). We retained environmental variables with correlation coefficients <0.8. After threshold‐based pre‐selection, eight environmental variables were included in the habitat suitability model, including mean diurnal range (Bio2), maximum temperature of the warmest month (Bio5), minimum temperature of warmest month (Bio6), temperature annual range (Bio7) annual precipitation (Bio12), precipitation of wettest month (Bio13), precipitation seasonality (Bio15), and NDVI based on the clustering relation of Pearson coefficient (Figure S1).

We used the eight environmental variables and 356 occurrence records as input data to predict the habitat suitability across the range of *B. gargarizans*. The predictive power of the habitat suitability model was evaluated using cross‐validation and receiver operating characteristic analysis (ROC) based on 20% of the occurrence localities (Phillips et al., 2006) and showed a good fit (AUC > 0.863). The habitat suitability values ranged from 0 to 1, with the highest values indicating the greatest habitat suitability. Figure S2 shows the predicted range of *B. gargarizans*. We divided landscape into a 500 × 500 m grid, and generated the resistance surface layer by assigning a habitat suitability value ranging from 0 to 1 to each grid cell in ArcMap 10.4 (ESRI). We used the resistance surface layer to calculate the least cost‐weighed distance (LCD) and circuit distance (CD).

### 2.5 Geographic distance

We used three pairwise distance matrices (Euclidean distance, LCD, and CD) to estimate the degree of geographic isolation between populations. Euclidean distance is by definition the straight‐line distance between localities without accounting for the effect of landscape characteristics. LCD and CD account for the heterogeneity of landscapes. LCD is the path that minimizes the total cumulative cost between two points through heterogeneous landscapes (Wang et al., 2009). CD is calculated by summarizing the costs of all possible paths between two points (McRae & Beier, 2007). CD accounts for factors such as species distributions and migration rates in heterogeneous landscapes by considering the relative resistance to different landscape features (McRae et al., 2008). We used the “spDists” function in the R package “sp” to calculate the Euclidean distance between populations (Pebesma & Bivand, 2005). We used Pathmatrix 1.1 in ARCVIEW 3.3 to calculate LCD between paired sites (McRae & Beier, 2007; Storfer et al., 2010), and Circuitscape 5.0 to calculate the pairwise CD (McRae & Beier, 2007). We used the eight‐neighbor connection scheme to calculate LCD and CD based on habitat suitability value for each grid cell.

### 2.6 Environmental distance

We used the same environmental variables (Bio2, Bio5, Bio6, Bio7, Bio12, Bio13, Bio15, and NDVI) in the habitat suitability model to estimate the environmental distance between populations. We used ArcMap 10.4 to extract values for every environmental variable of each population. We used R to calculate environmental distance by determining the absolute differences between populations and thereby produce eight pairwise environmental distance matrices (Wang et al., 2013).

### 2.7 Causes of genetic divergence

Genetic divergence was estimated by pairwise *F*_ST_ based on high‐quality SNPs across all populations in VCFtools. Genetic, environmental, and geographic distance matrices were obtained from the above methods. The Mantel test was used to determine the relationships between environmental/geographic and genetic distance matrices in the R package vegan. Spearman correlation coefficients were calculated, and two‐sided *p*‐values were calculated after performing 999 randomizations of the genetic distance matrix using the “mantel. test” function in the R package vegan.

We used reciprocal causal modeling (RCM) with partial Mantel tests, generalized dissimilarity modeling (GDM), and linear mixed effects models with maximum likelihood population effects (MLPE) to evaluate the effects of geographic distance and environmental dissimilarity on genetic differentiation. RCM was used to evaluate relationships between genetic distance and geographic distance and between environmental distance and geographic distance matrices while accounting for either environmental or geographic distance. A total of 110 tests were organized into 55 reciprocal causal models and the causal relationships were predicted following the methods of MacDonald et al. (2020). We firstly tested the partial Mantel relationship between genetic distance and two geographic/environmental distance as one reciprocal model in the R package vegan. We assessed the partial mantel's *R* coefficient (*R*_PM_) for partial Mantel test A (*R*_PM‐A_) and B (*R*_PM‐B_) between the genetic matrix and two geographic/environmental matrixes as reciprocal causal models. Comparing the values of *R*_PM‐A_ and *R*_PM‐B_, if *R*_PM‐A_ > *R*_PM‐B_, the variable form partial Mantel test A was better supported, and vice‐versa. A heatmap summarized results of *R*_PM‐A_ – *R*_PM‐B_.

GDM is a nonlinear extension of matrix regression that can model spatial variation in pairwise *F*_ST_ between localities caused by pairwise geographic and environmental differences. We performed GDM using the pairwise *F*_ST_ matrix for high quality SNP dataset as the response variable in the R package “gdm” 1.5.0 (Fitzpatrick et al., 2021). The maximum value of the fitted I‐splines was adjusted between 0 and 1 to assess the relative importance of different geographic and environmental variables used in the generalized dissimilarity model.

MLPE linear mixed effects can quantitatively analyze relationships between distance matrices while controlling for nonindependence among pairwise data (Shirk et al., 2017). We used the “MLPE.lmm” function within the R package “ResistanceGA” (Peterman, 2018) to perform MLPE linear mixed effects models to evaluate relationships between genetic distance and each of the 11 geographic and environmental variables. AIC scores were used to assess relative model support by setting REML = FALSE (Peterman, 2018; Shirk et al., 2017).

### 2.8 Genomic offset to future climate change

We used seven bioclimatic variables (Bio2, Bio5, Bio6, Bio7, Bio12, Bio13, and Bio15) derived from 2081 to 2100 to predict genomic offset at the population level. These future bioclimatic variables were derived from six global circulation models (GCMs; BBC‐CSM2‐MR, CanESM5, CNRM‐CM6‐1, CNRM‐ESM2‐1, MIROC6, and MIROC‐ES2L) and the shared socio‐economic pathway [SSP245 (an average scenario) for 2081–2100]. Future climate variables were obtained by extracting average values from the WorldClim database at a 2.5‐min spatial distribution (Fick & Hijmans, 2017). GDM was used to predict the genetic change (genetic offset sensu Fitzpatrick & Keller, 2015) needed to track a changing climate relative to future climate conditions under the SSP245 scenarios (Fick & Hijmans, 2017). We mapped the genomic offset to visualize the spatial distribution of population‐level vulnerability to climate change.

## 3 RESULTS

### 3.1 SNP genotyping, population genetic structure and diversity

We obtained 308,551 high‐quality SNPs from the 94 toads filtered by VCFtools. The number of SNPs was evenly distributed on the different chromosomes. The number of SNPs was lowest (5751) on chromosome 11 and highest (62,353) on chromosome 1 (Figure S3). A ML tree constructed using this subset of SNPs revealed that toads in our sample could be divided into three genetically distinct clusters: the western, northeastern, and central‐eastern clusters (Figure S4).

Pairwise *F*_ST_ values differed significantly from zero in most population pairs and ranged from 0.020 to 0.598, with an overall mean of 0.311 (Table S3). The results of the ADMIXTURE analysis were consistent with the topology of the ML tree; for K values from 2 to 10, the optimal K value was 3 for our 21 populations (Figure S5). The cross‐validation error for all the *K* values in the line plot suggested that the toads in our sample were first divided into two clusters by the boundary between the third and second terrain steps (the Daxinganling‐Taihang‐Wushan‐Xuefeng mountain ranges), and then the populations in the second terrain step (e.g., Yun‐Gui and Mongolia Plateau) were further divided into two clusters along the altitudinal gradient (Figure 1b). We constructed the plot of individual ancestry coefficients based on *K* = 3 in the PCA (Figure 1c). The results of the cluster analysis were consistent with those of the ADMIXTURE analysis. Strong geographic isolation was confirmed by the positive correlation between pairwise *F*_ST_ and Euclidean distance (*r* = 0.219, *p* = 0.017). A Mantel test revealed a positive correlation between pairwise *F*_ST_ with Bio5 (*r* = 0.587, *p* < 0.0001), NDVI (*r* = 0.229, *p* = 0.034) and longitude (*r* = 0.500, *p* < 0.0001). However, all other geographic and environmental variables were not correlated with pairwise *F*_ST_ (all *r* < 0.178 and all *p* > 0.075).

*H*_O_ did not differ from *H*_E_ within each of the 21 populations (*χ* ^2^ test, all *p* > 0.05; Table S1). Within‐population genetic variation was relatively low comparing with previous studies; mean ± SE for *H*_O_, *H*_E_, and π was 0.214 ± 0.014 (0.099–0.374), 0.452 ± 0.014 (0.370–0.614), and 0.083 ± 0.006 (0.040–0.123), respectively (Table S1). Thirteen populations showed high and significant *F*_IS_ values, ranging from 0.137 to 0.610 with a mean of 0.348. *H*_O_ was correlated negatively with Bio2, and positively with Bio6 (Table 1). However, *H*_E_ was correlated negatively with Bio2, Bio7 and Bio15, and positively correlated with Bio6, Bio12 and NDVI (Table 1). π was positively correlated with Bio5, and *F*_IS_ was negatively correlated with Bio7 (Table 1).

**TABLE 1:** Results of Pearson correlation analysis on the correlations between average observed heterozygosity (*H*_O_), average expected heterozygosity (*H*_E_), nucleotide diversity (π), or Wright's inbreeding coefficient (*F*_IS_) and environmental variables that are significant (*p* < 0.05).

Models	*r*	*t*	*p*
*H*_O_ ~ mean diurnal range (Bio2)	−0.473	2.338	0.030
*H*_O_ ~ minimum temperature of warmest month (Bio6)	0.468	2.307	0.032
*H*_E_ ~ mean diurnal range (Bio2)	−0.574	3.056	<0.01
*H*_E_ ~ minimum temperature of warmest month (Bio6)	0.604	3.300	<0.01
*H*_E_ ~ temperature annual range (Bio7)	−0.490	2.452	0.024
*H*_E_ ~ annual precipitation (Bio12)	0.446	2.172	0.043
*H*_E_ ~ precipitation seasonality (Bio15)	−0.566	2.996	<0.01
*H*_E_ ~ normalized difference vegetation index (NDVI)	0.454	2.219	0.039
π ~ maximum temperature of the warmest month (Bio5)	0.603	10.871	<0.01
*F*_IS_ ~ temperature annual range (Bio7)	−0.467	5.296	0.033

*Note*: The degree of freedom is 19 in all cases.

### 3.2 Environmental and geographic factors contributing to local adaptation

**FIGURE 2:**
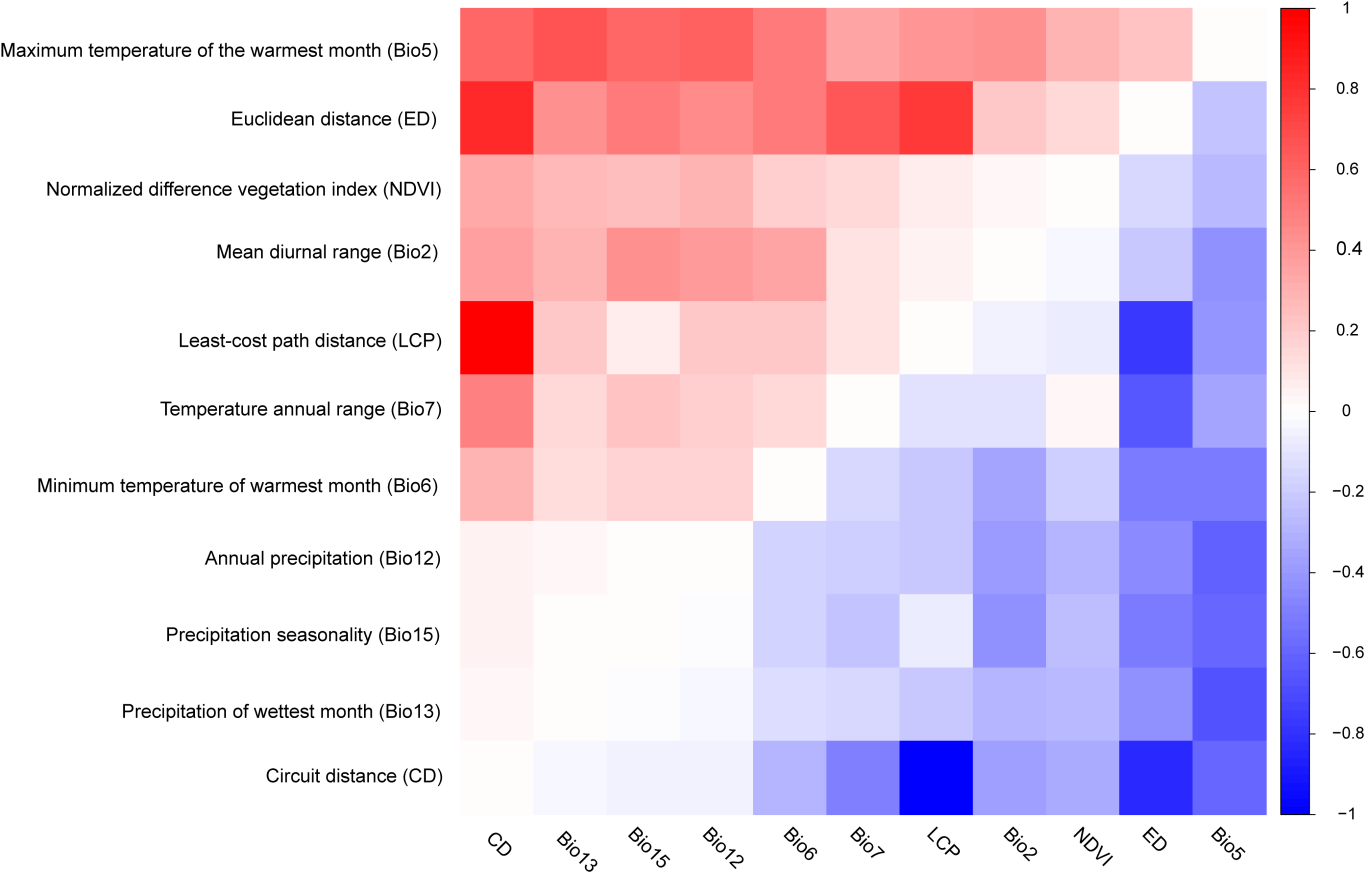
Pairwise visual heatmap showing reciprocal causal modeling results. Red and blue in individual cells respectively represent positive and negative values of the reciprocal model. The variables with more positive values have a stronger support.

Bio5 and Euclidean distance were the variables showing the strongest correlations with genetic distance after removing the effects of other variables (Figure 2). Euclidean distance was positively correlated with genetic distance after partialling out Bio5 (*R*_PM_ = 0.277, *p* = 0.009), and the reciprocal partial Mantel test indicated that genetic distance was positively correlated with Bio5 (*R*_PM_ = 0.512, *p* = 0.001). Moreover, partial Mantel tests revealed that there were nonsignificant relationships between genetic distance and geographic/environmental distances when either Bio5 or Euclidean distance (as alternative variables) was removed including LCD (*R*_PM_ = 0.148, *p* = 0.165), CD (*R*_PM_ = −0.016, *p* = 0.696), Bio2 (*R*_PM_ = 0.129, *p* = 0.151), Bio6 (*R*_PM_ = 0.064, *p* = 0.262), Bio12 (*R*_PM_ = −0.025, *p* = 0.572), Bio13 (*R*_PM_ = −0.084, *p* = 0.718), and Bio15 (*R*_PM_ = −0.015, *p* = 0.542). However, there were significant correlations between genetic distance and geographic/environmental distances after partialling out either Bio5 or Euclidean distance (as alternative variables) including NDVI (*R*_PM_ = 0.309, *p* = 0.005) and Bio7 (*R*_PM_ = 0.256, *p* = 0.023; Figure 2). Likewise, there were significant correlations between geographic/environmental distance and genetic distance when any environmental distance (as an alternative variable) was removed, indicating that genetic distance was affected by multiple environmental and geographic distances (Figure 2).

**FIGURE 3:**
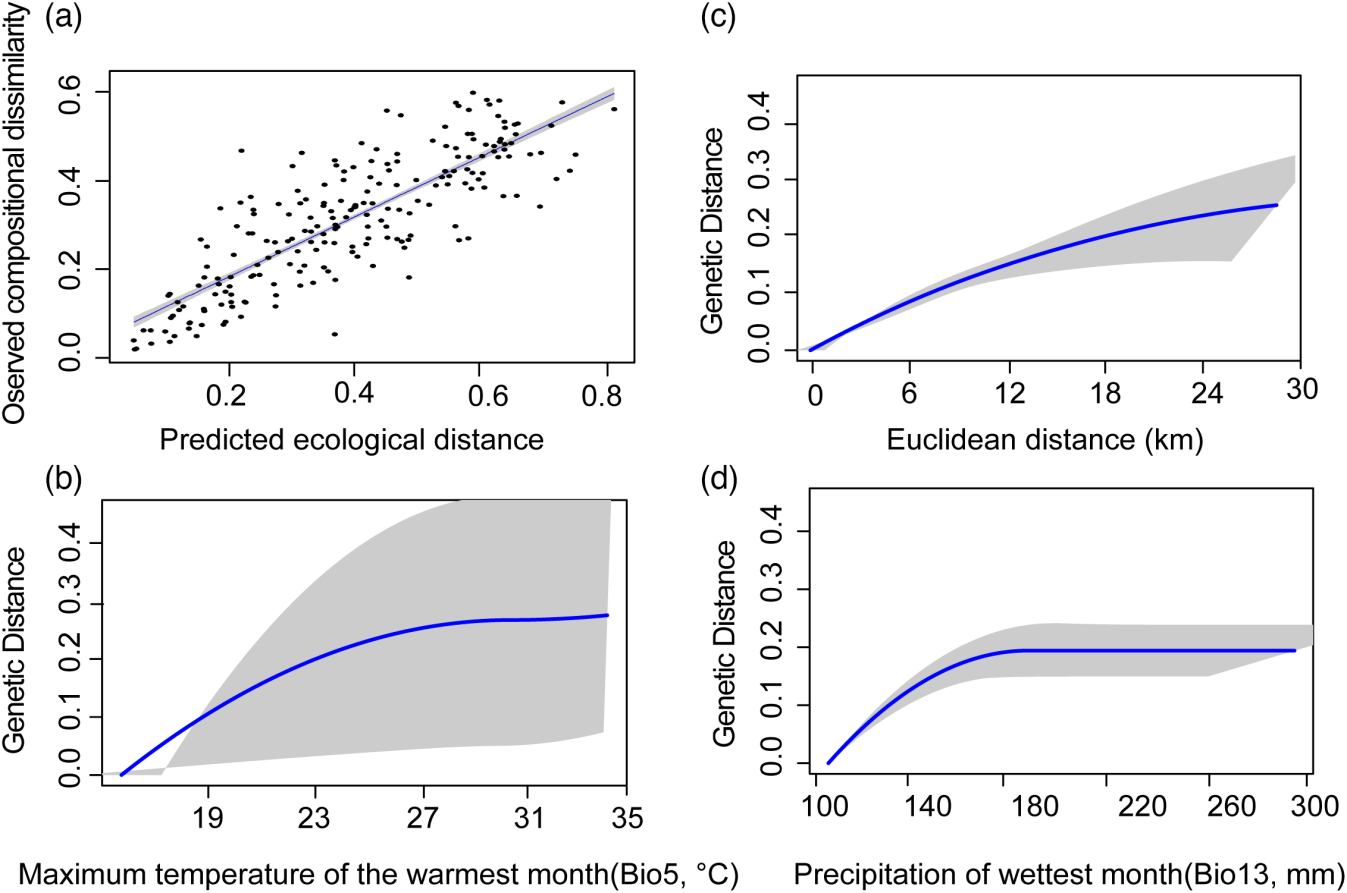
Fitted I‐splines of the generalized dissimilarity modelling for the relationship between observed compositional dissimilarity and predicted ecological distance (a), and the environmental correlates of partial ecological distance based on three important variables, maximum temperature of the warmest month (b), Euclidean distance (c) and precipitation of wettest month (d). Black dots indicate *F*_ST_ values between populations. The black line represents the model fit, and the shaded area is the error bands (±one standard deviation). Each curve indicates the relative importance of an environmental variable in explaining changes in allele frequency, holding all other variables constant. The shape of each curve shows the rate at which allele frequencies change along the gradient.

GDM explained 69.6% of the deviance in turnover in genetic composition (*p* < 0.0001). GDM showed that the best supported variables affecting genetic distances ranked in order of importance were Bio13 (*p* = 0.040), Euclidean distance (*p* < 0.0001) and Bio5 (*p* = 0.040; Figure 3). Results of MLPE linear mixed effects models showed that the best supported variable affecting genetic distance was the Bio12 matrix, followed by Euclidean distance, Bio5 and Bio13 matrixes (Table 2). The three modeling approaches (RCM, MLPE linear mixed effects models and GDM) indicated that geographic and environmental variables could explain the genetic variation observed in *B. gargarizans*. Therefore, population genetic structure in *B. gargarizans* is driven by a combination of geographic and environmental isolation.

**TABLE 2:** Relative support for the effects of geographic and environmental distances on genetic distance predicted using linear mixed effects models with maximum likelihood population effects.

Distance measure	AIC	ΔAIC
Annual precipitation (Bio12)	−241.97	0.00
Euclidean distance	−239.71	2.26
Maximum temperature of the warmest month (Bio5)	−239.52	2.45
Precipitation of wettest month (Bio13)	−238.53	3.44
Minimum temperature of warmest month (Bio6)	−238.74	3.50
Precipitation seasonality (Bio15)	−238.36	3.61
Temperature annual range (Bio7)	−237.68	4.29
Least‐cost path distance	−237.66	4.31
Mean diurnal range (Bio2)	−237.65	4.32
Circuit distance	−237.39	4.58
Normalized difference vegetation index (NDVI)	−237.26	4.70

### 3.3 Genomic offset to future climate warming

**FIGURE 4:**
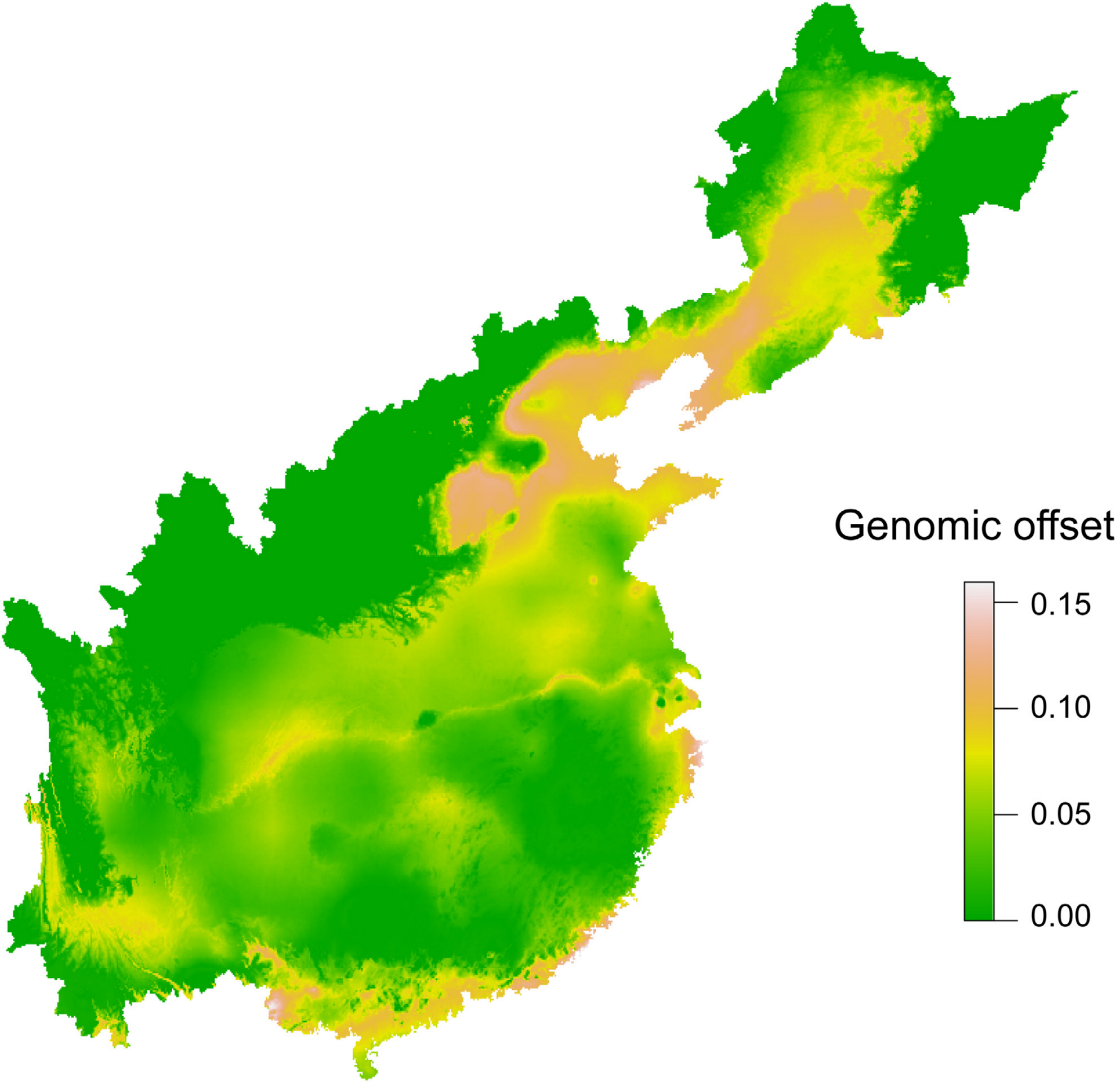
Climate change‐related genetic offset predicted from generalized dissimilarity modeling based on an average scenario of SSP245 in the 2100's climate. The color scale indicates the magnitude of the mismatch between current and future climate‐driven turnover of alleles, the more genomic offset to future climate conditions and higher risks of population decline.

The GDM model based on *F*_ST_ was predicted onto future climate conditions using the SSP245, resulting in an estimate of genetic differentiation between current populations and those adapted to the 2100's climate. The results showed the relatively large genetic change occurred in the high‐altitude populations (western populations), high‐latitude populations (northern populations) and populations along the Yangtze River and east coast (southeastern populations) compared with populations in the middle of the distributional range in China according to GDM analysis (Figure 4). Genomic offset estimates indicated that future climate change would increase the risk of extirpation in populations along the Yangtze River and in the western, northern and eastern coastal parts of the range of *B. gargarizans* in China (Figure 4).

## 4 DISCUSSION

### 4.1 Population structure

*Bufo gargarizans* in China has been divided into four (western, central, northeastern, and southeastern) clusters based on mtDNA, nuclear, and allozyme data of 166 specimens from 56 localities (Fu et al., 2005; Zhan & Fu, 2011) or seven clusters based on mtDNA data of 226 specimens from 57 localities (Tong et al., 2017). The geological uplift during the Pliocene, range expansion, and refugia in the western and southeastern regions of its range during the glacial periods have all contributed to genetic divergence within *B. gargarizans* (Fu et al., 2005; Zhan & Fu, 2011). A study of the meta‐population dynamics of *B. gargarizans* has shown that migrations from western China to other regions and from southern to northern China have occurred during the past ~10,000 years (Zhan & Fu, 2011). Our high‐quality SNP data revealed three genetically distinct clusters in the western, central‐eastern, and northeastern parts of mainland China (Figure 1). Our results indicated that the southeastern and central clusters formed a single cluster consisting of the southeastern and central populations that have often been reported in earlier studies (Figure S6; Fu et al., 2005). Our results also indicated that Asiatic toads expanded both from west to east and from south to north. This dispersal pattern is supported by the topology of the ML tree, which shows that the western and central‐southeastern clusters are ancestral to the northeastern cluster. Migration from west to northeast was not detected in this study. The mito‐nuclear discordance can be caused by various mechanisms, including nuclear introgression, sex‐biased asymmetries and irregular gene flow between populations (Funk & Omland, 2003; Stobie et al., 2018; Toews & Brelsford, 2012). Taken together, we tend to conclude that the complex topography, nuclear introgression and/or sex‐biased asymmetries all can be the potential main causes of mito‐nuclear discordance in *B. gargarizans*.

### 4.2 Population genetic diversity

The SNP‐based *F*_ST_ values among the populations in this study were higher than microsatellite‐based *F*_ST_ values among nine populations estimated using 111 samples (Wu & Hu, 2010), but lower than mtDNA control region‐based *F*_ST_ values among 33 populations estimated using 90 samples (Table S3; Fu et al., 2005). Differences in *F*_ST_ between mitochondrial and nuclear genes are common in the animal species, mainly due to their differences in evolutionary rates (Allio et al., 2017). *F*_ST_ was significantly correlated with Bio5 and geographic distance but not with other environmental or geographic variables, and genetic differentiation between populations increased with the maximum temperature of the warmest month and geographic distance. The results of Mantel tests suggested that geographic and environmental distances significantly affected genomic diversity. The correlations between *F*_ST_ and environmental matrices suggested local adaptation in the genome of *B. gargarizans*; similar results have been reported for other amphibians such as *Rana arvalis* (Rödin‐Mörch et al., 2021), *B. bufo* (Luquet et al., 2015), and *B. andrewsi* (Guo et al., 2016).

Genetic diversity increased or decreased significantly with climatic variables (e.g., *H*_O_/*H*_E_ vs. Bio2 and Bio6, *H*_O_ vs. Bio7, Bio12, Bio15 and NDVI, π vs. Bio5) among different populations. Genetic diversity is related to the evolutionary potential of species, their ability to respond to local and global environmental changes, and current population fitness (Reed & Frankham, 2003). Thus, populations of *B. gargarizans* that have experienced high degrees of climatic heterogeneity are expected to have increased survival potential compared with populations occurring in areas with more homogeneous climates. *F*_IS_ was inversely related to Bio7. High *F*_IS_ might result in genetic drift and ultimately local differentiation (Rai & Jain, 1982) and is associated with harsh environments (Cummins et al., 2019). The high *F*_IS_ of *B. gargarizans* populations in suitable environments suggests that the benefits of local adaptation, or possibly the costs of dispersal to or seeking mates in other environments, exceed the costs of inbreeding in *B. gargarizans*. Therefore, both climatic and geographic factors contribute to genetic diversity in the toad. Genetic diversity within species is of fundamental importance for organisms’ adaptation to future environmental changes and, consequently, for their long‐term survival.

### 4.3 Geographic and environmental isolation

The outputs of RCM and GDM models were consistent: the maximum temperature of the warmest month and Euclidian distance were the main drivers of the current pattern of genomic variation. Geographic and ecological factors have been shown to contribute to spatial genomic patterns in several species. For example, environmental variability has played a major role in shaping patterns of genetic differentiation in the long‐lived subalpine conifers (*Pinus cembra*; Tóth et al., 2019), terrestrial‐breeding frogs (Cummins et al., 2019), rainbowfishes (*Melanotaenia duboulayi*; Smith et al., 2020), and song sparrows (*Melospiza melodia*; Mikles et al., 2020).

IBD and IBE accounted for a large proportion of the spatial genomic variation in *B. gargarizans*, which is consistent with the results of an earlier study (Pan et al., 2018). Environmental and climatic variables can explain 69.6% of the deviance in turnover in genetic composition. The precipitation of wettest month (22.7%), Euclidean distance (12.6%), and maximum temperature of the warmest month (10.2%) accumulatively accounted for 45.5% of the variation. RCM revealed a significant correlation between Euclidean distance and genomic differentiation after the effect of temperature was removed. Moreover, a significant correlation between maximum temperature and genomic differentiation was observed after the effect of Euclidean distance was removed. Overall, these findings suggest that both environmental difference and Euclidian distance affect spatial patterns of genomic variation in *B. gargarizans*. IBD theory posits that genomic differences between populations can accumulate locally if dispersal is limited by geographic factors (Li et al., 2015; Sexton et al., 2014; Wang et al., 2013). However, IBE theory suggests that genetic differentiation increases with the magnitude of environmental differences among populations (MacDonald et al., 2020; Sexton et al., 2014). In our study, both IBE and IBD were the main drivers of spatial genomic differences, which were associated with adaptive differentiation and vicariance.

Previous studies on several species in East Asia have shown that the genomic patterns are driven by distinct mechanisms. For example, long‐term gene flow and population history have played more important roles than Pleistocene climate fluctuations in shaping spatial genetic structure in the wild boar *Sus scrofa* (Hu et al., 2020). However, the geological history and environmental heterogeneity of subtropical China have been the major drivers of genomic diversification and local adaptation in the rapid racerunner *Eremias velox* (Liu et al., 2019). Therefore, quantifying the relative contributions of IBD and IBE to the organism genomic evolution is helpful to determine whether organisms are impacted by local adaptation and to predict the effects of climate change on the distribution and survival of organisms in the future.

### 4.4 Adaptation to local environmental conditions

Both genomic diversity (e.g., *H*_E_ and π) and *F*_IS_ related to climatic variables were significantly correlated with climate, suggesting that the direction and strength of climate‐driven selection on populations of *B. gargarizans* vary throughout its range. Environmental variables have been reported to drive local adaptation in amphibians. For example, altitude drives variation in genomic diversity among Tibetan frog *(Nanorana parkeri*) populations (Wang et al., 2018). The unique climatic conditions of East Asia, including oxygen partial pressure (Niu et al., 2021), UV radiation (Norsang et al., 2011), and temperature (Muir et al., 2014), have favored the evolution of genes mediating local adaptation in many species. In this study, local environment significantly impacted the genetic diversity and shaped the genetic structure in *B. gargarizans*. The effects of local temperature and precipitation on amphibians have been demonstrated in many species, such as *P. guentheri* (Cummins et al., 2019) and *R. temporaria* (Muir et al., 2014). Furthermore, temperature and precipitation are the most significant factors that limit the distribution range of amphibians and drive the formation of genetic patterns of amphibians (Lawler et al., 2010; Muir et al., 2014). For example, changes in precipitation and temperature might affect the phenology, abundance, and performance of amphibians (Ficetola & Maiorano, 2016). Therefore, the influence of local environmental variables contributes to local adaptation in *B. gargarizans*.

### 4.5 Genomic offset to future climate change

This study and a previous one (Pan et al., 2018) both indicate that environmental factors affect the genomic population structure of *B. gargarizans*. RCM, GDM, and MLPE linear mixed effects models all indicated that environmental and geographic variables were the main factors affecting the spatial distribution of genomic variation in *B. gargarizans* (Figures 2 and 3). The genetic diversity within species can affect the ability of a species to withstand sudden changes in climate (Exposito‐Alonso et al., 2018). For example, central/eastern populations in the East Asian Tertiary relict *Euptelea* might be more vulnerable to extinction due to climate warming (Cao et al., 2020). GDM model analysis based on climate data at the end of the century and the genomic variances of *B. gargarizans* predicted that populations along the Yangtze River and in the western, northern and eastern coastal parts of the species' range might face higher climate change‐induced extirpation risk compared with other populations (Figure 4). Previous studies suggest that climate change (i.e., precipitation and temperature) increases the risk of local extirpation (Cummins et al., 2019; Mi et al., 2022). The slow rate at which physiological tolerances to heat evolve suggests that high temperature will be the main factor restricting the continued survival of species (Qu & Wiens, 2020). Complex landscapes mitigate the effects of higher temperatures to some extent but also result in increased fragmentation of populations in some areas (e.g., western populations). Thus, mitigating carbon emissions and slowing the pace of climate warming are important for protecting many species, including the Asiatic toad.

## 5 CONCLUSION

Population structure and genetic diversity analysis based on high‐quality SNPs revealed three clusters of *B. gargarizans* in the western, central‐eastern, and northeastern parts of the species' range in China. Asiatic toads may have experienced an expansion both from west to east and from south to north. Spatial genomic patterns in *B. gargarizans* were determined by the local environment and geographic distance, as revealed by the fact that both IBE and IBD were the main drivers of spatial genomic differences in *B. gargarizans*. Future global warming will increase the risk of extirpation in populations along the Yangtze River and in the western, northern, and eastern coastal parts of the range of *B. gargarizans* in China.

## CONFLICT OF INTEREST STATEMENT

The authors declare no conflicts of interest.

## Supporting information


Figure S1.

Figure S2.

Figure S3.

Figure S4.

Figure S5.

Figure S6.

Table S1.

Table S2.

Table S3.

## Data Availability

All raw sequences obtained in this study have been deposited in the National Genomics Data Center (NGDC) GSA database (accession number CRA005915). Supplemental tables and figures are available in the Supporting Information.
