# Supplementary figures and images for: Genomic insights into local adaptation in the Asiatic toad Bufo gargarizans, and its genomic offset to climate warming
Source: Evol Appl. 2023 May 2;16(5):1071–83. doi: 10.1111/eva.13555 (PMC10197391; doi:10.1111/eva.13555)

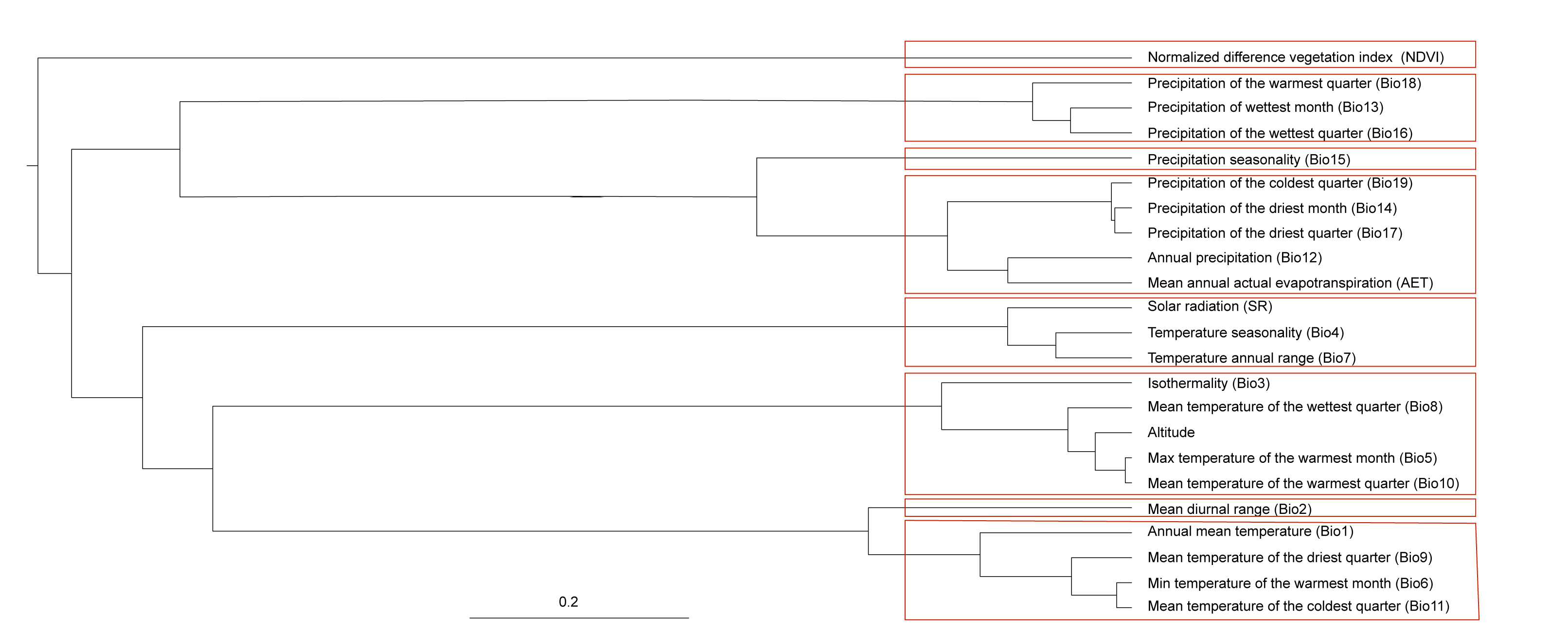

Supplement: Supplementary file 1 — Figure S1. [file EVA-16-1071-s001.tif]

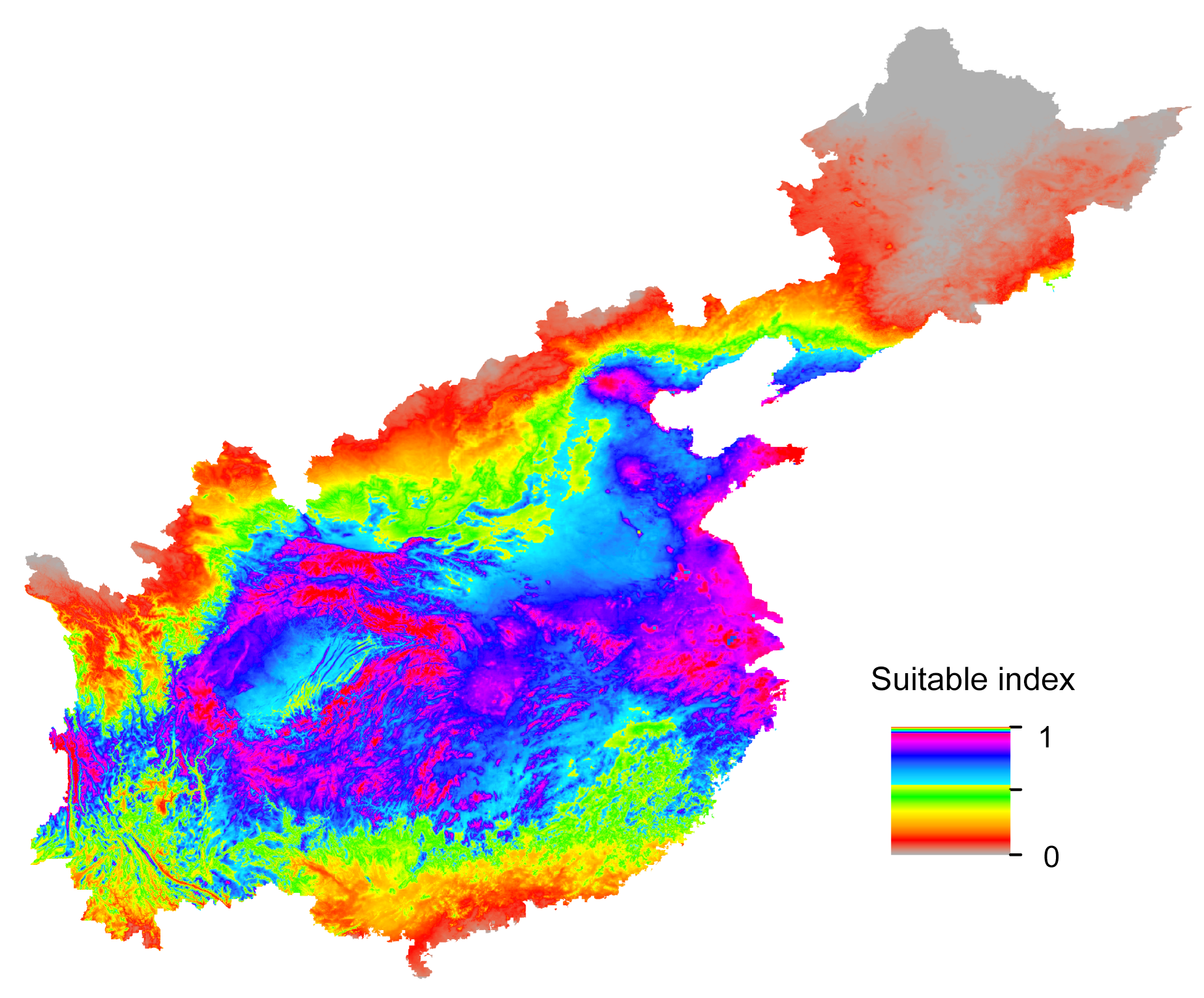

Supplement: Supplementary file 2 — Figure S2. [file EVA-16-1071-s003.tif]

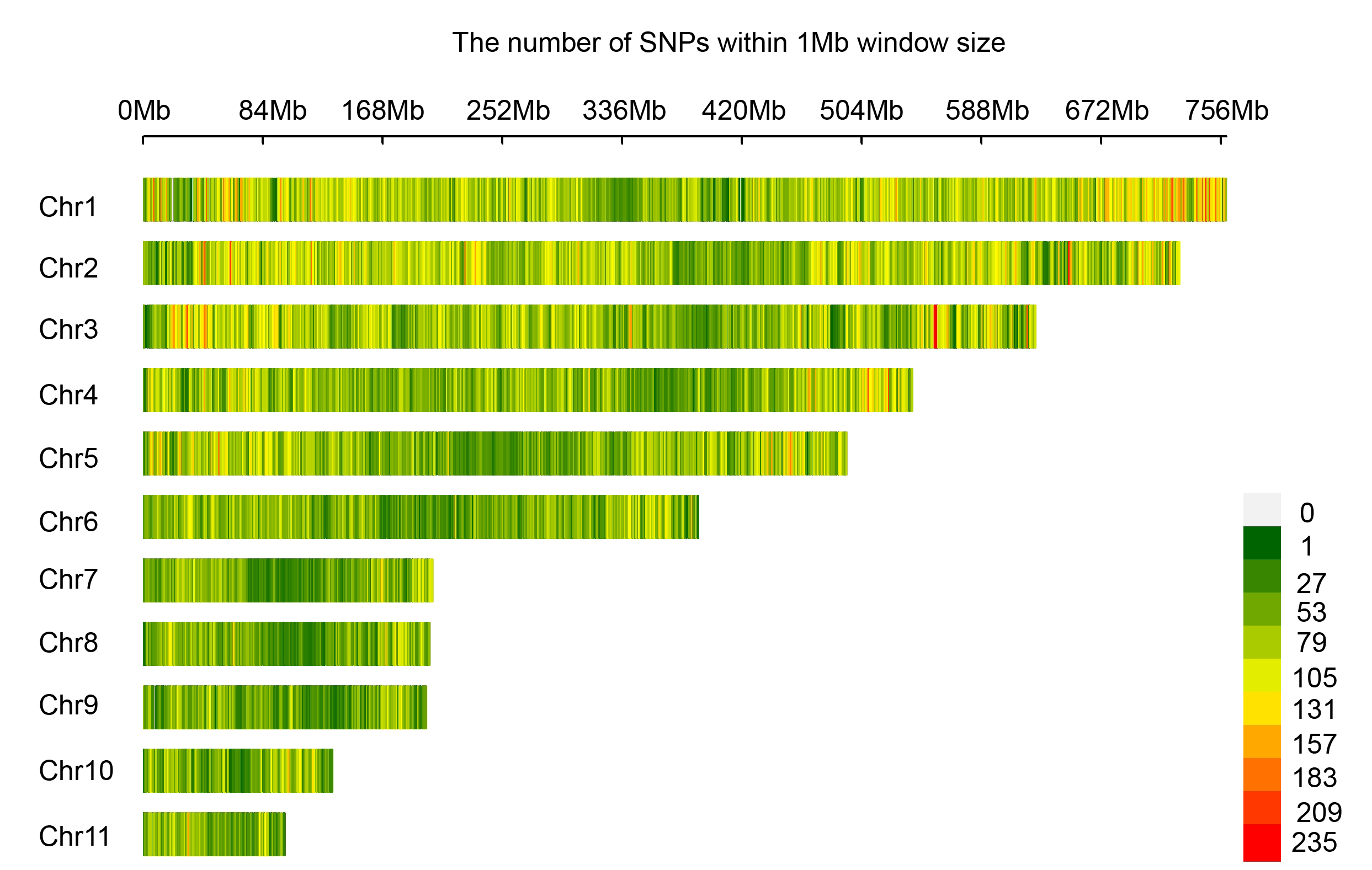

Supplement: Supplementary file 3 — Figure S3. [file EVA-16-1071-s006.tif]

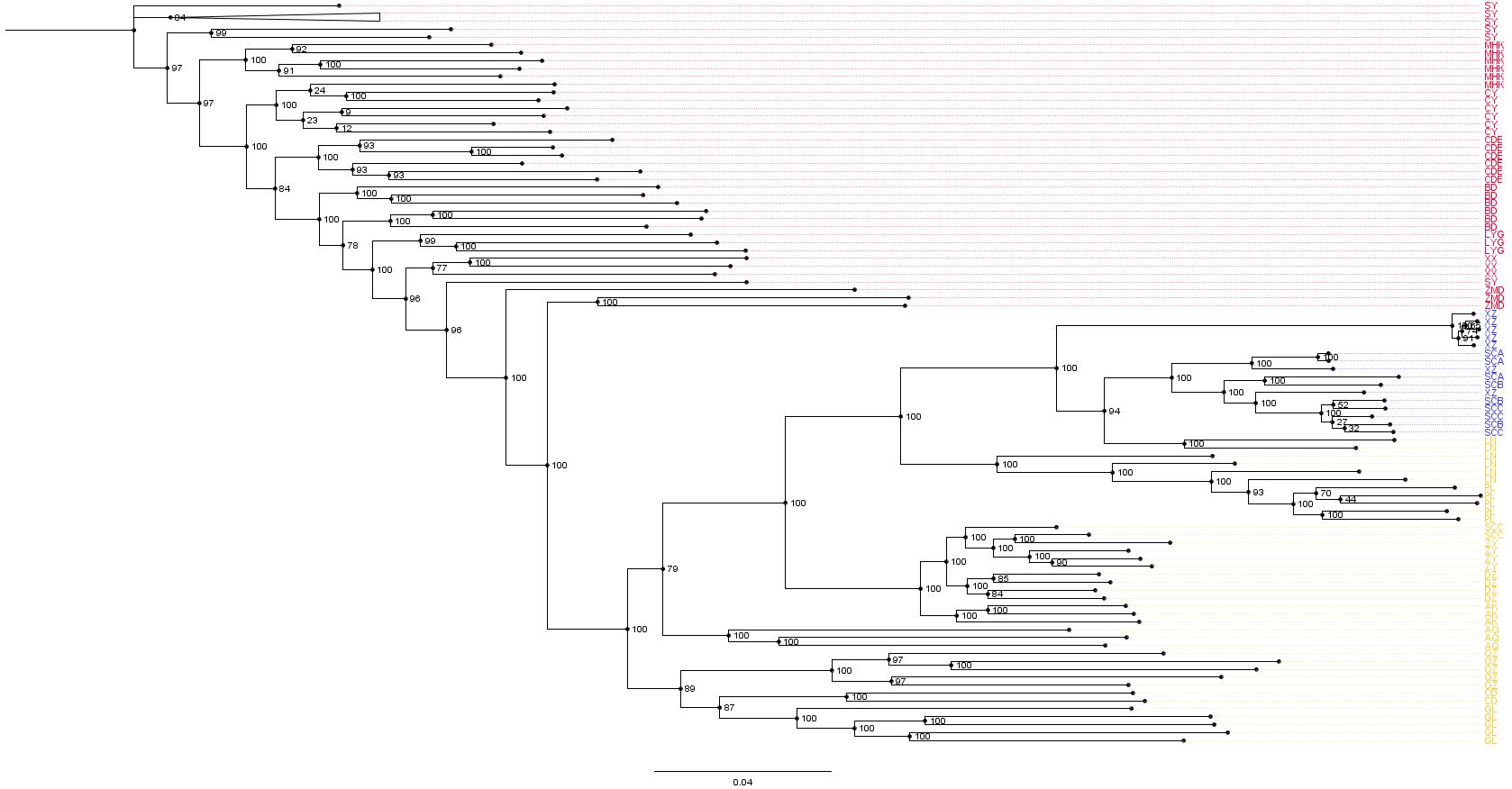

Supplement: Supplementary file 4 — Figure S4. [file EVA-16-1071-s007.tif]

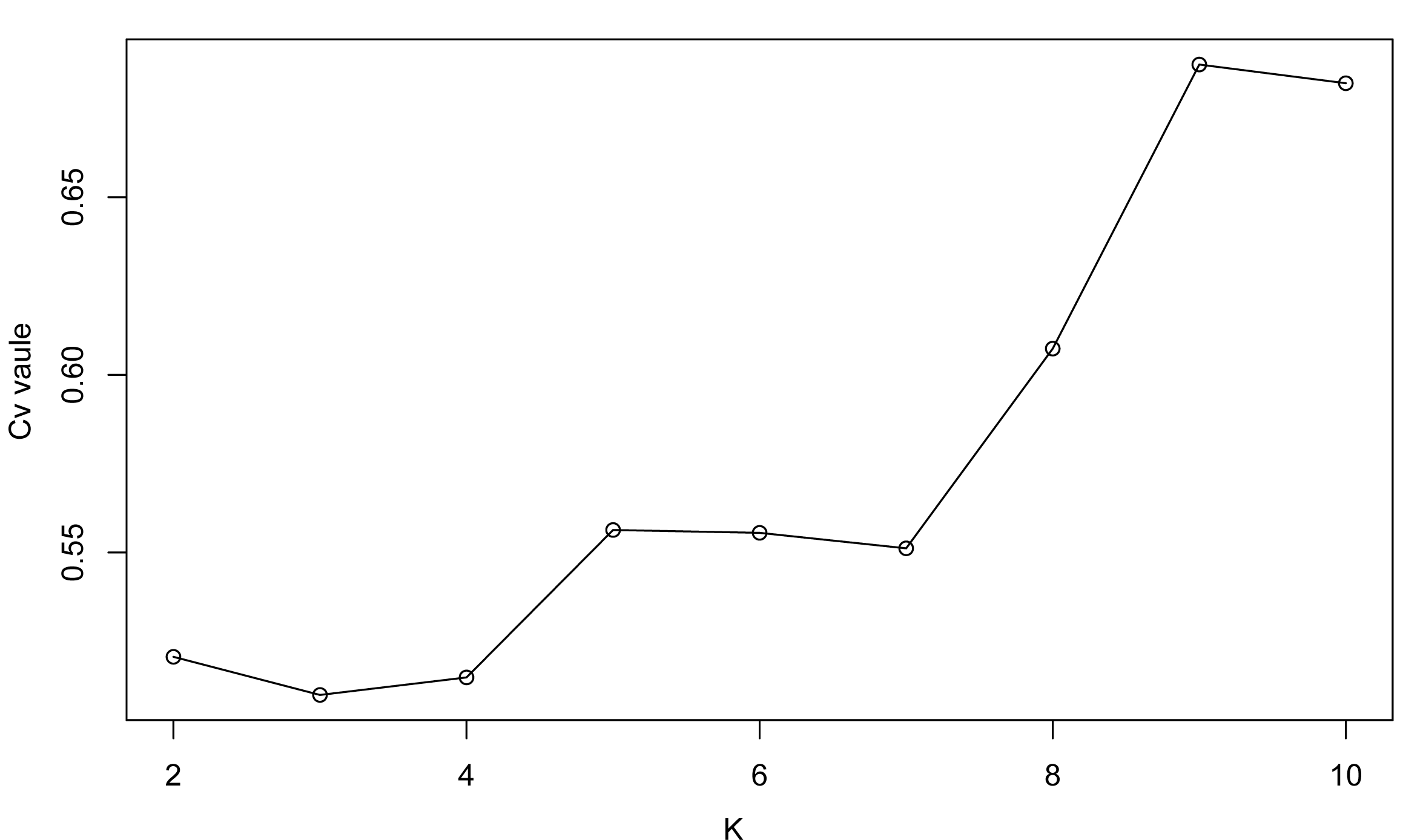

Supplement: Supplementary file 5 — Figure S5. [file EVA-16-1071-s005.tif]

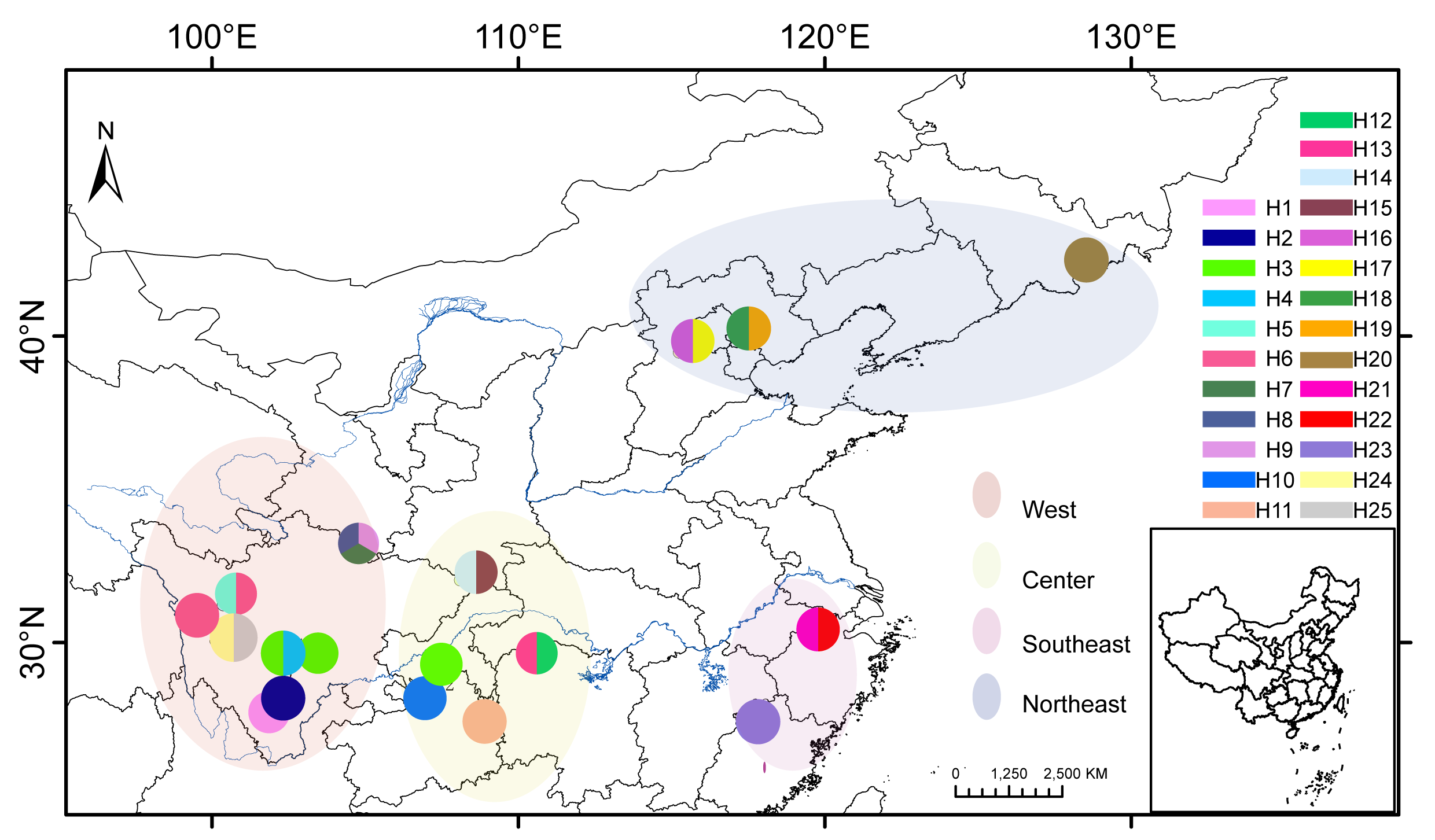

Supplement: Supplementary file 6 — Figure S6. [file EVA-16-1071-s004.tif]
