# Supplementary material for: Genomic insights into local adaptation in the Asiatic toad Bufo gargarizans, and its genomic offset to climate warming
Source: Evol Appl. 2023 May 2;16(5):1071–83. doi: 10.1111/eva.13555 (PMC10197391; doi:10.1111/eva.13555)
Supplement: Supplementary file 7 — Table S1. [file EVA-16-1071-s002.doc]

**Table S1** Information on sampling localities and genetic diversity statistics of *B. gargarizans* based on SNP markers.

| Locality | Longitude | Latitude | *N* | *H*_O_ | *H*_e_ | π | *F_is_* |
| --- | --- | --- | --- | --- | --- | --- | --- |
| Sichuan Province |  |  |  |  |  |  |  |
| Ganzi (BT) | 100.14 | 30.03 | 7 | 0.099 | 0.370 | 0.053 | 0.610 |
| Shimian (SCA) | 102.38 | 28.91 | 3 | 0.345 | 0.513 | 0.053 | 0.359 |
| Shimian (SCB) | 102.46 | 29.08 | 3 | 0.224 | 0.509 | 0.040 | 0.471 |
| Kangding (SCC) | 101.95 | 29.94 | 5 | 0.146 | 0.433 | 0.080 | 0.448 |
| Dazhou (DZ) | 106.94 | 30.80 | 4 | 0.212 | 0.452 | 0.047 | 0.438 |
| Gansu Province |  |  |  |  |  |  |  |
| Longnan (LN) | 105.22 | 33.90 | 6 | 0.165 | 0.399 | 0.097 | 0.315 |
| Pingliang (PL) | 106.16 | 35.36 | 5 | 0.189 | 0.410 | 0.058 | 0.289 |
| Guizhou Province |  |  |  |  |  |  |  |
| Zunyi (ZY) | 107.19 | 28.22 | 4 | 0.203 | 0.449 | 0.047 | 0.329 |
| Shanxi Province |  |  |  |  |  |  |  |
| Ankang (AK) | 108.91 | 32.35 | 3 | 0.253 | 0.503 | 0.064 | 0.299 |
| Anhui Province |  |  |  |  |  |  |  |
| Xiaoxian (XX) | 116.83 | 34.33 | 3 | 0.276 | 0.495 | 0.113 | 0.354 |
| Anqing (AQ) | 116.31 | 30.63 | 3 | 0.247 | 0.510 | 0.123 | 0.318 |
| Jiangsu Province |  |  |  |  |  |  |  |
| Lianyungang (LYG) | 119.15 | 34.33 | 3 | 0.245 | 0.523 | 0.095 | 0.252 |
| Henan Province |  |  |  |  |  |  |  |
| Zhumadian (ZMD) | 113.39 | 33.12 | 3 | 0.221 | 0.535 | 0.100 | 0.470 |
| Guangxi Province |  |  |  |  |  |  |  |
| Guilin (GL) | 110.89 | 25.90 | 5 | 0.193 | 0.420 | 0.119 | 0.352 |
| Zhejiang Province |  |  |  |  |  |  |  |
| Quzhou (QZ) | 118.55 | 28.91 | 5 | 0.208 | 0.416 | 0.119 | 0.365 |
| Hunan Province |  |  |  |  |  |  |  |
| Changde (CD) | 111.92 | 28.74 | 2 | 0.374 | 0.614 | 0.102 | 0.307 |
| Hebei Province |  |  |  |  |  |  |  |
| Baoding (BD) | 115.97 | 38.88 | 6 | 0.154 | 0.389 | 0.097 | 0.343 |
| Chengde (CDE) | 116.65 | 41.46 | 6 | 0.230 | 0.377 | 0.096 | 0.137 |
| Liaoning Province |  |  |  |  |  |  |  |
| Chaouyang (CY) | 120.44 | 41.55 | 6 | 0.201 | 0.376 | 0.092 | 0.196 |
| Shenyang (SY) | 123.45 | 41.89 | 6 | 0.153 | 0.391 | 0.084 | 0.342 |
| Jilin Province |  |  |  |  |  |  |  |
| Meihekou (MHK) | 125.86 | 42.67 | 6 | 0.154 | 0.398 | 0.080 | 0.322 |
