# Supplementary material for: Genomic insights into local adaptation in the Asiatic toad Bufo gargarizans, and its genomic offset to climate warming
Source: Evol Appl. 2023 May 2;16(5):1071–83. doi: 10.1111/eva.13555 (PMC10197391; doi:10.1111/eva.13555)
Supplement: Supplementary file 8 — Table S2. [file EVA-16-1071-s009.doc]

**Table S2 Geographic information on georeferenced occurrences of *B. gargarizans* from this study (see Table S1 for locality abbreviations) and the Global Biodiversity Information Facility (GBIF).**

| Number | Longitude | Latitude | Source |
| --- | --- | --- | --- |
| 1 | 100.745833 | 31.422778 | GBIF |
| 2 | 100.745833 | 31.425556 | GBIF |
| 3 | 100.141833 | 30.026461 | This study (BT) |
| 4 | 101.023056 | 27.848889 | GBIF |
| 5 | 101.231130 | 27.769030 | GBIF |
| 6 | 101.466420 | 29.179240 | GBIF |
| 7 | 101.500000 | 29.000000 | GBIF |
| 8 | 101.524180 | 31.096280 | GBIF |
| 9 | 101.570000 | 30.623611 | GBIF |
| 10 | 101.572500 | 30.409444 | GBIF |
| 11 | 101.604444 | 30.280556 | GBIF |
| 12 | 101.646840 | 28.636100 | GBIF |
| 13 | 101.703000 | 27.363710 | GBIF |
| 14 | 101.723140 | 28.691790 | GBIF |
| 15 | 101.838930 | 28.927340 | GBIF |
| 16 | 101.870000 | 30.270000 | GBIF |
| 17 | 101.947833 | 29.940461 | This study (SCC) |
| 18 | 102.100000 | 28.500000 | GBIF |
| 19 | 102.160203 | 27.922642 | GBIF |
| 20 | 102.179850 | 28.909520 | GBIF |
| 21 | 102.200000 | 28.910000 | GBIF |
| 22 | 102.216310 | 28.914290 | GBIF |
| 23 | 102.377833 | 28.908461 | This study (SCA) |
| 24 | 102.240000 | 29.800000 | GBIF |
| 25 | 102.250000 | 29.000000 | GBIF |
| 26 | 102.295556 | 29.087222 | GBIF |
| 27 | 102.309470 | 27.521770 | GBIF |
| 28 | 102.327000 | 27.880070 | GBIF |
| 29 | 102.377833 | 28.908461 | SCB |
| 30 | 102.406944 | 30.241944 | GBIF |
| 31 | 102.437744 | 30.152092 | GBIF |
| 32 | 102.480000 | 33.720000 | GBIF |
| 33 | 102.517410 | 27.868390 | GBIF |
| 34 | 102.557810 | 27.881010 | GBIF |
| 35 | 102.566667 | 27.883333 | GBIF |
| 36 | 102.592830 | 27.896160 | GBIF |
| 37 | 102.700000 | 25.000000 | GBIF |
| 38 | 102.700000 | 32.766667 | GBIF |
| 39 | 102.710000 | 30.680000 | GBIF |
| 40 | 102.710000 | 30.690000 | GBIF |
| 41 | 102.730000 | 24.270000 | GBIF |
| 42 | 102.736755 | 25.083856 | GBIF |
| 43 | 102.760990 | 28.533370 | GBIF |
| 44 | 102.763056 | 27.915833 | GBIF |
| 45 | 102.800000 | 30.300000 | GBIF |
| 46 | 102.810000 | 30.370000 | GBIF |
| 47 | 102.930000 | 30.550000 | GBIF |
| 48 | 102.930000 | 33.600000 | GBIF |
| 49 | 102.950000 | 30.550000 | GBIF |
| 50 | 102.95908 | 33.570410 | GBIF |
| 51 | 102.968056 | 29.680830 | GBIF |
| 52 | 102.968056 | 29.680833 | GBIF |
| 53 | 103.012470 | 29.750900 | GBIF |
| 54 | 103.051820 | 30.148170 | GBIF |
| 55 | 103.074380 | 30.186050 | GBIF |
| 56 | 103.120000 | 30.970000 | GBIF |
| 57 | 103.170000 | 31.030000 | GBIF |
| 58 | 103.180000 | 31.030000 | GBIF |
| 59 | 103.22095 | 30.784270 | GBIF |
| 60 | 103.230000 | 31.130000 | GBIF |
| 61 | 103.234167 | 30.870833 | GBIF |
| 62 | 103.240000 | 31.320000 | GBIF |
| 63 | 103.283333 | 29.583333 | GBIF |
| 64 | 103.300000 | 31.320000 | GBIF |
| 65 | 103.310000 | 31.080000 | GBIF |
| 66 | 103.334737 | 29.516007 | GBIF |
| 67 | 103.344633 | 29.928878 | GBIF |
| 68 | 103.350000 | 29.533333 | GBIF |
| 69 | 103.400000 | 29.600000 | GBIF |
| 70 | 103.490000 | 31.060000 | GBIF |
| 71 | 103.583333 | 31.466667 | GBIF |
| 72 | 103.590000 | 31.030000 | GBIF |
| 73 | 103.640000 | 32.800000 | GBIF |
| 74 | 103.730000 | 36.030000 | GBIF |
| 75 | 103.750000 | 31.233333 | GBIF |
| 76 | 103.816810 | 30.730190 | GBIF |
| 77 | 103.897310 | 31.356010 | GBIF |
| 78 | 104.000000 | 30.910000 | GBIF |
| 79 | 104.010000 | 30.910000 | GBIF |
| 80 | 104.069167 | 31.931389 | GBIF |
| 81 | 104.072300 | 30.630900 | GBIF |
| 82 | 104.088608 | 30.629990 | GBIF |
| 83 | 104.100000 | 32.970000 | GBIF |
| 84 | 104.100000 | 33.300000 | GBIF |
| 85 | 104.118000 | 30.645220 | GBIF |
| 86 | 104.150000 | 33.080000 | GBIF |
| 87 | 104.150100 | 30.567380 | GBIF |
| 88 | 104.412300 | 32.900000 | GBIF |
| 89 | 104.600000 | 31.000000 | GBIF |
| 90 | 104.745800 | 33.163330 | GBIF |
| 91 | 104.801500 | 32.565200 | GBIF |
| 92 | 105.000000 | 29.200000 | GBIF |
| 93 | 105.566700 | 32.300000 | GBIF |
| 94 | 105.603900 | 28.317180 | GBIF |
| 95 | 105.750000 | 30.583330 | GBIF |
| 96 | 105.215833 | 33.896461 | This study (LN) |
| 97 | 106.129600 | 28.664570 | GBIF |
| 98 | 106.200000 | 28.330000 | GBIF |
| 99 | 106.935833 | 30.800461 | This study (DZ) |
| 100 | 106.520000 | 32.630000 | GBIF |
| 101 | 106.550000 | 32.660000 | GBIF |
| 102 | 106.674100 | 26.430320 | GBIF |
| 103 | 107.008400 | 33.060830 | GBIF |
| 104 | 106.161833 | 35.358461 | This study (PL) |
| 105 | 107.195300 | 27.986200 | GBIF |
| 106 | 107.193833 | 28.220461 | This study (ZY) |
| 107 | 107.371900 | 29.088060 | GBIF |
| 108 | 107.437200 | 33.232900 | GBIF |
| 109 | 107.486800 | 33.651050 | GBIF |
| 110 | 107.881300 | 26.381900 | GBIF |
| 111 | 108.913833 | 32.348461 | This study (AK) |
| 112 | 108.955000 | 28.097780 | GBIF |
| 113 | 109.058500 | 32.675330 | GBIF |
| 114 | 109.114400 | 34.015560 | GBIF |
| 115 | 109.114400 | 34.016670 | GBIF |
| 116 | 110.031000 | 29.693090 | GBIF |
| 117 | 110.891833 | 25.898461 | This study (GL) |
| 118 | 110.175700 | 25.238540 | GBIF |
| 119 | 110.243100 | 24.173370 | GBIF |
| 120 | 110.400000 | 29.300000 | GBIF |
| 121 | 110.427000 | 29.312570 | GBIF |
| 122 | 110.492400 | 29.023420 | GBIF |
| 123 | 110.501300 | 24.771610 | GBIF |
| 124 | 110.506800 | 29.374490 | GBIF |
| 125 | 110.800000 | 30.100000 | GBIF |
| 126 | 113.385833 | 33.122461 | This study (ZMD) |
| 127 | 112.339000 | 27.984700 | GBIF |
| 128 | 112.428400 | 29.739770 | GBIF |
| 129 | 112.550000 | 29.820000 | GBIF |
| 130 | 112.806300 | 24.936120 | GBIF |
| 131 | 112.917500 | 24.980830 | GBIF |
| 132 | 111.923833 | 28.736461 | This study (CD) |
| 133 | 113.034300 | 24.951900 | GBIF |
| 134 | 113.035700 | 24.893330 | GBIF |
| 135 | 113.073600 | 28.079010 | GBIF |
| 136 | 113.247100 | 25.284440 | GBIF |
| 137 | 113.372200 | 23.040540 | GBIF |
| 138 | 113.452100 | 28.547980 | GBIF |
| 139 | 113.889700 | 22.896360 | GBIF |
| 140 | 114.032400 | 25.832680 | GBIF |
| 141 | 114.110900 | 24.542080 | GBIF |
| 142 | 114.123600 | 30.560830 | GBIF |
| 143 | 114.128600 | 30.562220 | GBIF |
| 144 | 114.135300 | 30.566670 | GBIF |
| 145 | 114.138100 | 30.565830 | GBIF |
| 146 | 114.483500 | 38.115810 | GBIF |
| 147 | 114.506700 | 30.409170 | GBIF |
| 148 | 114.507800 | 30.419720 | GBIF |
| 149 | 114.511400 | 30.412190 | GBIF |
| 150 | 114.512200 | 30.414720 | GBIF |
| 151 | 114.512500 | 30.412780 | GBIF |
| 152 | 114.512800 | 30.410830 | GBIF |
| 153 | 114.512800 | 30.413060 | GBIF |
| 154 | 114.513100 | 30.410560 | GBIF |
| 155 | 114.513600 | 30.409720 | GBIF |
| 156 | 114.514200 | 30.409170 | GBIF |
| 157 | 115.410600 | 39.801940 | GBIF |
| 158 | 115.589600 | 39.640590 | GBIF |
| 159 | 115.800000 | 28.700000 | GBIF |
| 160 | 116.309833 | 30.628461 | This study (AQ) |
| 161 | 115.965833 | 38.884461 | This study (BD) |
| 162 | 116.161700 | 40.059700 | GBIF |
| 163 | 116.163500 | 40.177280 | GBIF |
| 164 | 116.167000 | 40.053160 | GBIF |
| 165 | 116.184500 | 39.986060 | GBIF |
| 166 | 116.261300 | 39.994940 | GBIF |
| 167 | 116.367000 | 23.525910 | GBIF |
| 168 | 116.377200 | 40.017050 | GBIF |
| 169 | 116.389200 | 40.016550 | GBIF |
| 170 | 116.428000 | 26.299010 | GBIF |
| 171 | 116.46410 | 39.909990 | GBIF |
| 172 | 116.653833 | 41.464461 | This study (CDE) |
| 173 | 116.825833 | 34.326461 | This study (XX) |
| 174 | 116.970300 | 36.748330 | GBIF |
| 175 | 116.970300 | 36.749170 | GBIF |
| 176 | 116.970300 | 36.749440 | GBIF |
| 177 | 116.970600 | 36.748330 | GBIF |
| 178 | 116.970600 | 36.749720 | GBIF |
| 179 | 116.972500 | 36.750250 | GBIF |
| 180 | 116.978500 | 36.748140 | GBIF |
| 181 | 116.981100 | 36.747500 | GBIF |
| 182 | 116.983600 | 36.742380 | GBIF |
| 183 | 116.985000 | 36.740000 | GBIF |
| 184 | 116.989200 | 36.723610 | GBIF |
| 185 | 117.076200 | 27.007170 | GBIF |
| 186 | 117.080600 | 27.004130 | GBIF |
| 187 | 117.157500 | 34.215850 | GBIF |
| 188 | 117.191900 | 32.260880 | GBIF |
| 189 | 117.200000 | 40.10000 | GBIF |
| 190 | 117.419200 | 31.743060 | GBIF |
| 191 | 117.424700 | 31.740560 | GBIF |
| 192 | 117.436800 | 40.649120 | GBIF |
| 193 | 117.438900 | 31.714170 | GBIF |
| 194 | 117.463700 | 40.642350 | GBIF |
| 195 | 117.575300 | 31.635000 | GBIF |
| 196 | 117.575600 | 31.635280 | GBIF |
| 197 | 117.581100 | 31.636390 | GBIF |
| 198 | 117.581400 | 31.636670 | GBIF |
| 199 | 117.582500 | 31.637220 | GBIF |
| 200 | 117.627500 | 31.715690 | GBIF |
| 201 | 117.637500 | 31.716570 | GBIF |
| 202 | 117.660000 | 27.741390 | GBIF |
| 203 | 117.693600 | 27.760310 | GBIF |
| 204 | 118.000000 | 27.700000 | GBIF |
| 205 | 118.170000 | 36.180000 | GBIF |
| 206 | 118.545833 | 28.908461 | This study (QZ) |
| 207 | 118.327500 | 29.726850 | GBIF |
| 208 | 118.345000 | 31.886940 | GBIF |
| 209 | 118.380000 | 30.200000 | GBIF |
| 210 | 118.380000 | 31.330000 | GBIF |
| 211 | 118.591400 | 32.089440 | GBIF |
| 212 | 118.591700 | 32.085840 | GBIF |
| 213 | 118.594400 | 32.081390 | GBIF |
| 214 | 118.595000 | 32.080280 | GBIF |
| 215 | 118.595600 | 32.079720 | GBIF |
| 216 | 118.595600 | 32.090830 | GBIF |
| 217 | 118.596700 | 32.078610 | GBIF |
| 218 | 118.604200 | 32.081110 | GBIF |
| 219 | 118.612800 | 31.610000 | GBIF |
| 220 | 118.614700 | 31.614410 | GBIF |
| 221 | 118.615600 | 31.61528 | GBIF |
| 222 | 118.616100 | 31.614720 | GBIF |
| 223 | 118.616700 | 31.615560 | GBIF |
| 224 | 118.619200 | 31.616580 | GBIF |
| 225 | 118.773300 | 31.958020 | GBIF |
| 226 | 118.774400 | 31.923610 | GBIF |
| 227 | 118.780300 | 31.931110 | GBIF |
| 228 | 118.780600 | 31.931110 | GBIF |
| 229 | 118.780800 | 31.933060 | GBIF |
| 230 | 118.780800 | 31.934170 | GBIF |
| 231 | 118.780800 | 31.934440 | GBIF |
| 232 | 118.781900 | 31.935000 | GBIF |
| 233 | 118.782500 | 31.935000 | GBIF |
| 234 | 118.782500 | 31.935280 | GBIF |
| 235 | 118.782800 | 31.935280 | GBIF |
| 236 | 118.788200 | 32.074360 | GBIF |
| 237 | 118.811800 | 32.075930 | GBIF |
| 238 | 118.816100 | 32.056110 | GBIF |
| 239 | 118.817200 | 32.056390 | GBIF |
| 240 | 118.817500 | 32.056390 | GBIF |
| 241 | 118.817800 | 32.056390 | GBIF |
| 242 | 118.817800 | 32.056670 | GBIF |
| 243 | 118.81790 | 32.060390 | GBIF |
| 244 | 118.8181000 | 32.054440 | GBIF |
| 245 | 118.818100 | 32.054720 | GBIF |
| 246 | 118.818100 | 32.055000 | GBIF |
| 247 | 118.818100 | 32.056670 | GBIF |
| 248 | 118.818300 | 32.056520 | GBIF |
| 249 | 118.818300 | 32.056520 | GBIF |
| 250 | 118.818300 | 32.056530 | GBIF |
| 251 | 118.818400 | 32.056510 | GBIF |
| 252 | 118.818600 | 32.056390 | GBIF |
| 253 | 118.818900 | 32.056390 | GBIF |
| 254 | 118.819100 | 32.056370 | GBIF |
| 255 | 118.820000 | 32.057220 | GBIF |
| 256 | 118.820000 | 32.057780 | GBIF |
| 257 | 118.824400 | 31.758890 | GBIF |
| 258 | 118.825300 | 31.756110 | GBIF |
| 259 | 118.826700 | 31.756110 | GBIF |
| 260 | 118.826900 | 31.756110 | GBIF |
| 261 | 118.830000 | 31.761110 | GBIF |
| 262 | 118.831100 | 31.761670 | GBIF |
| 263 | 118.832200 | 31.761940 | GBIF |
| 264 | 118.841400 | 31.753610 | GBIF |
| 265 | 118.842200 | 31.754440 | GBIF |
| 266 | 118.846300 | 31.758530 | GBIF |
| 267 | 118.903900 | 31.872220 | GBIF |
| 268 | 118.904200 | 31.871940 | GBIF |
| 269 | 118.908900 | 31.870010 | GBIF |
| 270 | 118.920000 | 31.850830 | GBIF |
| 271 | 118.927100 | 31.848620 | GBIF |
| 272 | 119.455800 | 30.337650 | GBIF |
| 273 | 119.482400 | 30.187350 | GBIF |
| 274 | 119.527500 | 31.176580 | GBIF |
| 275 | 119.535800 | 29.890560 | GBIF |
| 276 | 119.677800 | 29.800830 | GBIF |
| 277 | 119.678100 | 29.800830 | GBIF |
| 278 | 119.678600 | 29.673890 | GBIF |
| 279 | 119.678900 | 29.801110 | GBIF |
| 280 | 119.679200 | 29.800830 | GBIF |
| 281 | 119.679400 | 29.801110 | GBIF |
| 282 | 119.679400 | 29.801670 | GBIF |
| 283 | 119.693200 | 29.093720 | GBIF |
| 284 | 119.700000 | 30.200000 | GBIF |
| 285 | 119.716000 | 29.770680 | GBIF |
| 286 | 119.737200 | 30.213560 | GBIF |
| 287 | 119.848300 | 31.069740 | GBIF |
| 288 | 119.848900 | 31.069080 | GBIF |
| 289 | 119.851900 | 31.066940 | GBIF |
| 290 | 119.852200 | 31.066940 | GBIF |
| 291 | 119.854200 | 31.065830 | GBIF |
| 292 | 119.855300 | 31.064720 | GBIF |
| 293 | 119.855800 | 31.064440 | GBIF |
| 294 | 119.855800 | 31.065560 | GBIF |
| 295 | 119.856100 | 31.064170 | GBIF |
| 296 | 119.857200 | 31.053060 | GBIF |
| 297 | 119.858300 | 31.062780 | GBIF |
| 298 | 119.859700 | 31.061950 | GBIF |
| 299 | 119.867000 | 31.066890 | GBIF |
| 300 | 119.147833 | 34.3264610 | This study (LYG) |
| 301 | 120.157300 | 33.365160 | GBIF |
| 302 | 120.394200 | 27.521800 | GBIF |
| 303 | 120.463900 | 33.134170 | GBIF |
| 304 | 120.558700 | 36.333860 | GBIF |
| 305 | 120.662900 | 36.236580 | GBIF |
| 306 | 120.728700 | 31.156970 | GBIF |
| 307 | 120.730000 | 31.599810 | GBIF |
| 308 | 120.732400 | 31.600220 | GBIF |
| 309 | 120.737100 | 31.607340 | GBIF |
| 310 | 120.761100 | 30.712500 | GBIF |
| 311 | 120.763900 | 31.594630 | GBIF |
| 312 | 120.764200 | 30.721670 | GBIF |
| 313 | 120.768100 | 30.715280 | GBIF |
| 314 | 120.835900 | 32.867020 | GBIF |
| 315 | 120.898000 | 31.418960 | GBIF |
| 316 | 121.086400 | 29.724430 | GBIF |
| 317 | 121.099500 | 31.278020 | GBIF |
| 318 | 121.137100 | 31.276450 | GBIF |
| 319 | 121.169500 | 31.274490 | GBIF |
| 320 | 121.194600 | 31.762620 | GBIF |
| 321 | 121.222400 | 31.045320 | GBIF |
| 322 | 121.369300 | 31.111130 | GBIF |
| 323 | 121.423300 | 31.024540 | GBIF |
| 324 | 121.448300 | 31.149580 | GBIF |
| 325 | 121.471800 | 31.187150 | GBIF |
| 326 | 121.494800 | 31.251490 | GBIF |
| 327 | 121.497100 | 32.059010 | GBIF |
| 328 | 121.523200 | 31.173320 | GBIF |
| 329 | 121.563300 | 31.229710 | GBIF |
| 330 | 121.592200 | 37.323210 | GBIF |
| 331 | 121.648300 | 31.050290 | GBIF |
| 332 | 121.862300 | 29.840390 | GBIF |
| 333 | 123.241500 | 42.679370 | GBIF |
| 334 | 123.321500 | 42.741010 | GBIF |
| 335 | 120.437833 | 41.550461 | This study (CY) |
| 336 | 123.590300 | 41.831110 | GBIF |
| 337 | 123.590300 | 41.831940 | GBIF |
| 338 | 123.590300 | 41.835000 | GBIF |
| 339 | 123.447833 | 41.894461 | This study (SY) |
| 340 | 123.908600 | 41.381820 | GBIF |
| 341 | 125.855833 | 42.668461 | This study (HEB) |
| 342 | 126.481100 | 45.784380 | GBIF |
| 343 | 126.630000 | 45.750000 | GBIF |
| 344 | 127.583500 | 45.365720 | GBIF |
| 345 | 128.089400 | 42.308680 | GBIF |
| 346 | 128.311900 | 42.736390 | GBIF |
| 347 | 99.200000 | 28.700000 | GBIF |
| 348 | 99.210000 | 27.330000 | GBIF |
| 349 | 99.220000 | 27.330000 | GBIF |
| 350 | 99.300000 | 27.550000 | GBIF |
| 351 | 99.360000 | 27.650000 | GBIF |
| 352 | 99.420000 | 27.580000 | GBIF |
| 353 | 99.460000 | 27.570000 | GBIF |
| 354 | 99.700000 | 27.700000 | GBIF |
| 355 | 99.719070 | 28.023070 | GBIF |
| 356 | 99.720560 | 30.991670 | GBIF |
