# Supplementary material for: Genomic insights into local adaptation in the Asiatic toad Bufo gargarizans, and its genomic offset to climate warming
Source: Evol Appl. 2023 May 2;16(5):1071–83. doi: 10.1111/eva.13555 (PMC10197391; doi:10.1111/eva.13555)
Supplement: Supplementary file 9 — Table S3. [file EVA-16-1071-s008.doc]

**Table S3** Pairwise *F_ST_* (below the diagonal) and geographical distance (above the diagonal, in km) for 21 populations of *B. gargarizans*. See Table S1 for locality abbreviations.

| Pop | BT | SCA | SCB | SCC | LN | PL | DZ | ZY | AK | AQ | LYG | XX | ZMD | GL | QZ | CD | BD | CDE | CY | MHK | SY |
| --- | --- | --- | --- | --- | --- | --- | --- | --- | --- | --- | --- | --- | --- | --- | --- | --- | --- | --- | --- | --- | --- |
| BT | 0.00 | 248.58 | 247.06 | 174.13 | 659.71 | 651.00 | 829.10 | 707.99 | 883.09 | 1675.86 | 1557.96 | 1891.19 | 1320.68 | 1132.15 | 1776.15 | 1143.31 | 1814.09 | 2035.82 | 2336.64 | 2603.03 | 2846.82 |
| SCA | 0.33 | 0.00 | 20.88 | 122.15 | 491.03 | 619.63 | 806.26 | 474.99 | 742.35 | 1529.97 | 1369.55 | 1740.01 | 1169.49 | 893.78 | 1573.78 | 929.40 | 1726.24 | 1969.85 | 2250.27 | 2508.27 | 2750.23 |
| SCB | 0.34 | 0.23 | 0.00 | 107.98 | 474.80 | 598.58 | 785.15 | 469.49 | 724.51 | 1512.66 | 1356.49 | 1723.06 | 1152.61 | 892.17 | 1562.93 | 920.10 | 1705.88 | 1948.78 | 2230.14 | 2487.66 | 2729.51 |
| SCC | 0.32 | 0.19 | 0.05 | 0.00 | 490.04 | 540.98 | 726.51 | 540.45 | 722.65 | 1514.26 | 1385.97 | 1727.58 | 1157.52 | 971.96 | 1603.42 | 970.52 | 1677.35 | 1910.47 | 2200.17 | 2461.41 | 2703.69 |
| LN | 0.35 | 0.27 | 0.27 | 0.17 | 0.00 | 381.45 | 512.19 | 287.94 | 255.58 | 1022.75 | 895.53 | 1230.52 | 667.97 | 663.23 | 1128.67 | 528.81 | 1245.74 | 1505.86 | 1758.38 | 2002.24 | 2237.64 |
| PL | 0.57 | 0.57 | 0.58 | 0.42 | 0.14 | 0.00 | 184.53 | 657.02 | 382.26 | 1072.64 | 1086.51 | 1286.77 | 758.96 | 1032.17 | 1349.56 | 844.11 | 1136.69 | 1350.06 | 1642.63 | 1903.31 | 2140.21 |
| DZ | 0.58 | 0.58 | 0.60 | 0.21 | 0.30 | 0.52 | 0.00 | 799.21 | 417.50 | 973.85 | 1059.96 | 1183.21 | 700.70 | 1136.00 | 1332.52 | 902.90 | 971.69 | 1168.88 | 1466.32 | 1727.86 | 1962.22 |
| ZY | 0.56 | 0.56 | 0.58 | 0.18 | 0.30 | 0.53 | 0.11 | 0.00 | 488.97 | 1162.58 | 932.44 | 1653.79 | 815.58 | 444.91 | 1114.87 | 466.97 | 1464.50 | 1740.07 | 1969.98 | 2201.82 | 2433.98 |
| AK | 0.55 | 0.55 | 0.56 | 0.21 | 0.26 | 0.49 | 0.12 | 0.18 | 0.00 | 775.10 | 720.61 | 986.21 | 428.82 | 740.89 | 982.35 | 491.19 | 983.38 | 1247.46 | 1489.59 | 1729.38 | 1962.07 |
| AQ | 0.46 | 0.43 | 0.42 | 0.27 | 0.26 | 0.41 | 0.35 | 0.35 | 0.25 | 0.00 | 413.92 | 213.23 | 343.09 | 1084.06 | 622.82 | 767.46 | 512.94 | 793.87 | 869.06 | 1038.24 | 1244.20 |
| LYG | 0.52 | 0.51 | 0.51 | 0.33 | 0.32 | 0.47 | 0.45 | 0.44 | 0.35 | 0.16 | 0.00 | 492.77 | 393.93 | 738.49 | 286.97 | 469.44 | 918.62 | 1205.36 | 1277.09 | 1426.81 | 1620.68 |
| XX | 0.42 | 0.45 | 0.45 | 0.30 | 0.29 | 0.43 | 0.39 | 0.39 | 0.30 | 0.13 | 0.02 | 0.00 | 545.79 | 1205.42 | 604.99 | 909.08 | 585.03 | 826.09 | 811.96 | 929.56. | 1113.49 |
| ZMD | 0.49 | 0.48 | 0.47 | 0.28 | 0.27 | 0.44 | 0.38 | 0.38 | 0.27 | 0.08 | 0.10 | 0.06 | 0.00 | 836.18 | 671.17 | 506.35 | 684.27 | 976.24 | 1144.35 | 1352.57 | 1573.31 |
| GL | 0.42 | 0.39 | 0.38 | 0.27 | 0.28 | 0.40 | 0.34 | 0.34 | 0.27 | 0.13 | 0.20 | 0.16 | 0.14 | 0.00 | 835.57 | 332.03 | 1530.58 | 1824.30 | 1985.16 | 2177.40 | 2391.17 |
| QZ | 0.40 | 0.37 | 0.37 | 0.27 | 0.27 | 0.39 | 0.35 | 0.35 | 0.28 | 0.15 | 0.21 | 0.18 | 0.17 | 0.15 | 0.00 | 644.87 | 1137.35 | 1408.26 | 1417.74 | 1520.77 | 1687.41 |
| CD | 0.52 | 0.53 | 0.53 | 0.33 | 0.31 | 0.49 | 0.47 | 0.46 | 0.36 | 0.12 | 0.23 | 0.19 | 0.14 | 0.13 | 0.16 | 0.00 | 1195.25 | 1488.53 | 1649.03 | 1844.76 | 2060.37 |
| BD | 0.21 | 0.41 | 0.42 | 0.29 | 0.29 | 0.40 | 0.36 | 0.35 | 0.28 | 0.17 | 0.04 | 0.03 | 0.09 | 0.20 | 0.21 | 0.21 | 0.00 | 293.00 | 487.55 | 728.99 | 728.99 |
| CDE | 0.48 | 0.46 | 0.46 | 0.34 | 0.33 | 0.43 | 0.40 | 0.40 | 0.34 | 0.24 | 0.13 | 0.12 | 0.18 | 0.26 | 0.26 | 0.30 | 0.07 | 0.00 | 315.45 | 568.13 | 778.36 |
| CY | 0.49 | 0.47 | 0.47 | 0.34 | 0.33 | 0.44 | 0.41 | 0.41 | 0.35 | 0.36 | 0.12 | 0.11 | 0.18 | 0.25 | 0.47 | 0.30 | 0.06 | 0.05 | 0.00 | 253.38 | 467.69 |
| MHK | 0.51 | 0.48 | 0.48 | 0.35 | 0.34 | 0.46 | 0.42 | 0.42 | 0.36 | 0.32 | 0.11 | 0.11 | 0.18 | 0.26 | 0.26 | 0.30 | 0.08 | 0.09 | 0.06 | 0.00 | 217.10 |
| SY | 0.49 | 0.45 | 0.45 | 0.32 | 0.32 | 0.44 | 0.39 | 0.39 | 0.32 | 0.21 | 0.08 | 0.08 | 0.15 | 0.30 | 0.24 | 0.261 | 0.05 | 0.08 | 0.04 | 0.022 | 0.000 |
